# Network meta-analysis of robotic ventral and incisional hernia repair techniques: intraperitoneal, preperitoneal, retromuscular, and sublay approaches

**DOI:** 10.1007/s10029-026-03828-y

**Published:** 2026-10-01

**Authors:** Ibrahim Moqbel, Mohamed Basyouni Helal, Ahmed Hamada Abourageh, Joe C. Elhage, Ahmed A. Lashin, Amna Mutasim Elazrag, Karim Kamal Ahmed, Mohamed Nabil Galal, Masoud Altabawi, Ivan Capobianco

**Affiliations:** 1 Faculty of Medicine, Cairo University, Cairo, Egypt; 2 Faculty of Medicine, Menoufia University, Menoufia, Egypt; 3 Faculty of Medicine, Alexandria University, Alexandria, Egypt; 4 Faculty of Medicine, American University of Beirut, Beirut, Lebanon; 5 Faculty of Medicine, Benha University, Qalyubia, Egypt; 6 Faculty of Medicine, University of Khartoum, Khartoum, Sudan; 7 Faculty of Medicine, Nahda University in Beni Suef, Beni Suef, Egypt; 8 Faculty of Medicine, Tripoli University, Tripoli, Libya; 9 Department of General, Visceral and Transplant Surgery, University Hospital Tübingen, Tübingen, Germany

**Keywords:** Ventral hernia repair, Incisional hernia, Robotic surgery, IPOM, IPOM-plus, TAPP, TEP-RM, TA-RM, TAR, RS, TA-SM, TARM, ETEP, ETAPP, PP, Minimally invasive surgery, Network meta-analysis

## Abstract

**Objective:**

To compare the efficacy and safety of robotic ventral and incisional hernia repair techniques using network meta-analysis.

**Background:**

Robotic ventral hernia repair continues to evolve, but the comparative performance of individual robotic approaches remains uncertain.

**Methods:**

We conducted a systematic review and network meta-analysis of 14 studies, including 2 randomized trials and 12 retrospective cohorts, involving 9,348 adults undergoing ventral or incisional hernia repair. Techniques were classified by mesh plane and operative access. Frequently reported approaches were analyzed as separate nodes, whereas less commonly described but conceptually similar techniques were grouped into pooled preperitoneal and retromuscular nodes. Treatment effects were estimated using a R (netmeta); heterogeneity, transitivity were assessed, and certainty was rated with CINeMA.

**Results:**

rIPOM and rTAPP ranked highest for operative efficiency (SUCRA 94% and 96%, respectively), whereas rTAR ranked lowest. Compared with rIPOM, overall complications were lower with rTEP-RM (RR 0.23, 95% CI 0.07–0.74) and rTAPP (RR 0.55, 95% CI 0.30–0.99). rTEP-RM was also associated with lower seroma rates (RR 0.28, 95% CI 0.10–0.80) and shorter length of stay (MD − 5.33 days, 95% CI − 9.08 to − 1.58), although postoperative pain was higher. Surgical site infection was lower with rRS (RR 0.11, 95% CI 0.02–0.87) and rTAPP (RR 0.32, 95% CI 0.13–0.78). Pooled preperitoneal approaches were associated with lower short-term recurrence and 30-day readmission.

**Conclusions:**

Preperitoneal approaches, particularly rTEP-RM and rTAPP, were associated with more favorable early postoperative outcomes than rIPOM, although certainty of evidence was mostly low or very low.

**Supplementary Information:**

The online version contains supplementary material available at https://doi.org/10.1007/s10029-026-03828-y.

## Introduction

A hernia is defined as the protrusion of a portion of peritoneal sac, with or without an organ or portion thereof, through a defect in the abdominal wall. Wang et al. reported that the global burden of hernia disease increased by 36% between 1990 and 2019 [1]. Ventral hernias (VH), comprising spigelian, epigastric, umbilical, and incisional hernias (IH), are frequent complications after abdominal wall surgery and may seriously reduce the patients’ quality of life owing to the bulging, pain, feeling of discomfort, and cosmetic problems [2].

Historically, open surgery represented the gold standard for ventral hernia repair (VHR). However, this approach presented substantial drawbacks, including prolonged hospitalization, compromised cosmetic outcomes, and increased operative morbidity [3]. LeBlanc and Booth reported the first laparoscopic ventral hernia repair in 1991 [4]. This minimally invasive approach achieved superior cosmetic results, lower surgical site infection rates, and reduced recovery times. However, laparoscopy carries a greater risk of iatrogenic bowel injury than open techniques [5]. Capoccia Giovannini et al. [6] demonstrated that robotic-assisted ventral hernia repair (RVHR) was superior to open techniques and comparable in efficacy to laparoscopic approaches [6].

Nonetheless, the optimal robotic approach remains uncertain because direct randomized comparisons are scarce, operative nomenclature is inconsistent, and technique selection is strongly influenced by hernia morphology and reconstructive complexity. In the Intra-Peritoneal Onlay Mesh (IPOM) technique, mesh is put in position intraperitoneally without closing the fascial defect, whereas the defect is closed before mesh insertion in the IPOM-plus technique [2]. Placement of a retromuscular mesh is performed in both the Enhanced-view Totally Extra-Peritoneal (eTEP) and the Trans-Abdominal Retro-Muscular (TARM) techniques. In comparison, eTEP demonstrates superior outcomes relative to TARM, with lower complication rates and reduced length of hospital stay (LOS) [7]. A characteristic of yet another method, Trans-Abdominal Pre-Peritoneal (TAPP), is that it is often performed robotically [2].

We therefore performed a network meta-analysis of comparative studies to map the current evidence base across robotic techniques. Because randomized head-to-head data are limited, comparative observational studies were included to better characterize available signals and evidence gaps, while all estimates were interpreted cautiously in light of likely confounding and low certainty. Network meta-analysis enables simultaneous ranking of competing techniques and exploration of their relative safety and efficacy [8]. Accordingly, we conducted a network meta-analysis to compare the effectiveness and safety profiles of the following robotic ventral hernia repair techniques: rIPOM, rTAPP, rTEP-RM, rTAR, rRM, rTA-RM, rRS, PP (preperitoneal group), RM (retromuscular group), eTEP, eTAPP, TARM, and TA-SM.

## Methods

This systematic review was conducted in accordance with the Cochrane Handbook for Systematic Reviews of Interventions [9]. Reporting followed the Preferred Reporting Items for Systematic Reviews and Meta-Analyses (PRISMA) 2020 guidelines [10]. The study protocol was prospectively registered with the International Prospective Register of Systematic Reviews (PROSPERO) under the (CRD420251020252).

### Information sources, search strategy, and definition of intervention nodes

Search Strategy — A detailed search strategy was developed collaboratively with surgical and methodological experts. We conducted a search of the following electronic databases: PubMed/MEDLINE, Embase, Scopus, Web of Science and Cochrane Central Register of Controlled Trials (CENTRAL), from conception until May 2025. The search terms consisted of both controlled vocabulary (e.g., MeSH) and free-text keywords used in various combinations focusing on robotic ventral hernia repair, rIPOM, rTAPP, rTEP-RM, rTAR, rRM, rTA-RM, rRS, PP (preperitoneal group), RM (retromuscular group), eTEP, eTAPP, TARM, and TA-SM. We also searched for the reference list of included studies and other reviews for additional eligible records. Conference proceedings and trial registries (eg, ClinicalTrials.gov) were also searched to reduce the risk of publication bias. The full electronic search strategies for all databases are provided in Supplementary Table 5.

Predetermined inclusion criteria were selected. The population included adult patients (aged ≥ 18 years) undergoing primary or recurrent ventral or incisional hernia repair. For network construction, interventions were classified according to final mesh plane and operative access. Named techniques reported consistently across studies were retained as separate nodes only when sufficient data were available (e.g., rIPOM, rTAPP, rTEP-RM, rTAR, rRS), whereas conceptually similar but sparsely represented approaches were collapsed into higher-order anatomic nodes (preperitoneal [PP] and retromuscular [RM]) to limit node proliferation and preserve network connectivity. Comparisons at the level of mesh plane — particularly preperitoneal versus intraperitoneal placement — represent the most robust and clinically interpretable estimates supported by this network; comparisons between individual named techniques are reported as exploratory, hypothesis-generating signals that are necessarily informed by sparser data. The PP node included preperitoneal mesh-placement approaches not retained as separate named nodes, and the RM node included retromuscular approaches not retained as separate named nodes. We recognize that these pooled nodes may still contain clinically heterogeneous techniques that differ in access route, case selection, and reconstructive complexity; therefore, comparisons involving PP or RM should be interpreted with particular caution. Detailed study-to-node mapping is provided in Supplementary Table 4. Outcomes were selected a priori and defined according to the prespecified outcome definitions described below.

Inclusion criteria were: (1) publications in English; (2) randomized controlled trials (RCTs), non-randomized controlled trials, observational studies, cohort studies, and comparative studies (prospective or retrospective); and (3) adult populations regardless of baseline hernia characteristics. Studies were required to report at least one outcome of interest specified in the PICO framework. Exclusion criteria were: (1) non-English publications; (2) case reports, case series with fewer than 10 patients, reviews, and guidelines; (3) in vitro or animal studies; and (4) studies not directly addressing the PICO question. Potential overlap among cohorts was assessed by comparing author groups, institutions, enrollment periods, procedural descriptions, and baseline characteristics. When overlap was suspected, we retained the most informative dataset for the relevant comparison and outcome. Nevertheless, because several reports originated from the same investigative groups and reporting was incomplete in some studies, residual overlap cannot be fully excluded and may have influenced the precision of some network estimates.

### Screening and data management

The screening process was performed in sequential stages. Title and abstract screening included 10,621 records, randomly divided between two teams of three reviewers for independent dual assessment, with conflicts resolved by a third reviewer. Subsequently, 431 articles underwent full-text review in two rounds, with studies distributed among reviewer pairs for independent assessment. Any disagreements were resolved by discussion with a third independent reviewer.

### Data extraction

Data extraction was independently performed by two reviewers per study using a standardized form to minimize errors and ensure consistency. Extracted data encompassed: (1) study characteristics (author, publication year, country, study design, sample size); (2) patient demographics and hernia characteristics; (3) detailed surgical descriptions; and (4) follow-up duration. Data regarding the outcomes of interest were based on definitions outlined in the PICO framework and consistent with the focus of the review. Consensus of the discrepancies were obtained from those differences between pairs of reviewers, to ensure reliability and precision in extracted data. When agreement could not be reached, a third reviewer served as arbiter for final decisions.

### Outcome definitions and time-point selection

Primary outcomes were hernia recurrence, overall complications, surgical site infection, and reoperation. Secondary outcomes included operative time, postoperative pain, hospital stay, intraoperative complications, seroma, hematoma, and 30-day readmission. Outcomes were extracted using the original study definitions when standardized criteria were not available. Hernia recurrence was analyzed as a follow-up-dependent outcome. For each study, we extracted the recurrence estimate at the time point closest to 12 months only when recurrence ascertainment methods and follow-up completeness were sufficiently reported. Studies limited to 30-day or very short-term follow-up, or those with unclear recurrence ascertainment, were not considered primary sources for the main recurrence interpretation. Accordingly, recurrence findings should be interpreted as comparative signals from heterogeneous follow-up frameworks rather than definitive estimates of long-term durability. See Supplementary Table 3.

### Risk of bias assessment

Two independent reviewers assessed risk of bias using domain-specific tools. For randomized controlled trials, we applied the Cochrane Risk of Bias 2 (RoB 2) [11] tool; for non-randomized studies, we used the ROBINS-I [12] (Risk of Bias in Non-Randomized Studies of Interventions) tool. Disagreements were resolved through discussion to reach consensus. All included studies underwent rigorous quality assessment prior to quantitative synthesis.

### Publication bias

For outcomes reported in 10 or more studies, publication bias was assessed using funnel plot inspection for asymmetry and small-study effects. Egger’s regression test was additionally used to quantitatively evaluate evidence of funnel plot asymmetry, thus objectively assessing publication bias.

### Choice of the meta-analysis model

Given anticipated clinical heterogeneity, we used an outcome-specific modeling strategy and assessed heterogeneity with I^2^ and τ^2^. Common-effect models were used when heterogeneity was low and transitivity was plausible, while random-effects models were applied when heterogeneity was greater or more conservative estimation was warranted. We then performed a frequentist network meta-analysis to compare multiple robotic techniques, including indirect comparisons, and ranked treatments using SUCRA values.

### Calculation of missing data

When standard deviations were not reported, we imputed these values using standard formulas from the Cochrane Handbook to enable study inclusion. Where imputation was insufficient, we contacted study authors directly to obtain missing outcome data. All imputations and author-provided data are transparently reported.

### Statistical analysis

All statistical analyses were conducted using R software with the netmeta package for network meta-analysis [13, 14]. We used a frequentist approach to estimate treatment effects and compare the efficacy of multiple robotic techniques. In contrast to traditional pairwise meta-analyses, this framework permits simultaneous estimation of direct and indirect comparisons across multiple interventions. Multi-arm studies were incorporated using network methods that preserve within-study correlation among comparisons. Incoherence between direct and indirect evidence was assessed using local and global diagnostics. The accuracy of the overall network evidence for the effect of this specific treatment was improved by including head-to-head studies comparing one treatment to another. Continuous variables were analyzed using mean differences (MD) or standardized mean differences (SMD) as appropriate, while dichotomous variables were analyzed using risk ratios (RR). 95% CI was used to predict statistical significance. The interval containing 0 for MD and 1 for RR indicated no significant difference. Heterogeneity was evaluated by a Chi-squared test, with significant heterogeneity defined as P < 0.1 and I2 ≥ 50%. A random effects model was utilized to account for the heterogeneity in the analysis. To rank the interventions based on their effectiveness for each outcome, we used the surface under cumulative ranking (SUCRA) approach. The SUCRA approach has a value index that begins at 0% (least effective) and ends at 100% (most effective). This approach provides a comparative measure of intervention performance [15]. Due treatment ranking metrics may be unstable in sparse networks and do not incorporate clinical context directly, SUCRA values were interpreted as exploratory summaries rather than definitive indicators of clinical superiority.

### Clinical heterogeneity and transitivity

In addition to statistical heterogeneity, clinical transitivity was assessed by comparing key effect modifiers across treatment nodes, including age, BMI, hernia type, defect width, mesh dimensions, reconstructive complexity, and defect closure status. Defect closure data were extracted from all 14 studies. Among studies reporting arm-level rates, rIPOM showed variable closure rates (60–88%), whereas preperitoneal and retromuscular approaches achieved closure rates of 92–100%. In studies without numerical data, defect closure was considered mandatory when described as an integral component of the extraperitoneal technique. Accordingly, the rIPOM node was characterised predominantly as IPOM-plus, although some bridged repairs were present. Potential residual confounding related to differential closure rates, particularly for seroma, recurrence, and overall complications, was considered when interpreting the network estimates (supplementary Table 6).

### Assessment of certainty of evidence

Certainty of evidence for each network estimate was assessed with CINeMA, based on the GRADE approach for network meta-analysis [16, 17]. Two independent reviewers evaluated six domains: within-study bias, reporting bias, indirectness, imprecision, heterogeneity, and incoherence. Risk of bias was assessed using RoB 2 for randomized trials and ROBINS-I for non-randomized studies. Reporting bias was examined with funnel plots and Egger’s test when at least 10 studies were available. Indirectness, imprecision, heterogeneity, and incoherence were assessed using standard CINeMA criteria. See Tables S1 and S2.

## Results

### Search and screening

**Fig. 1:**
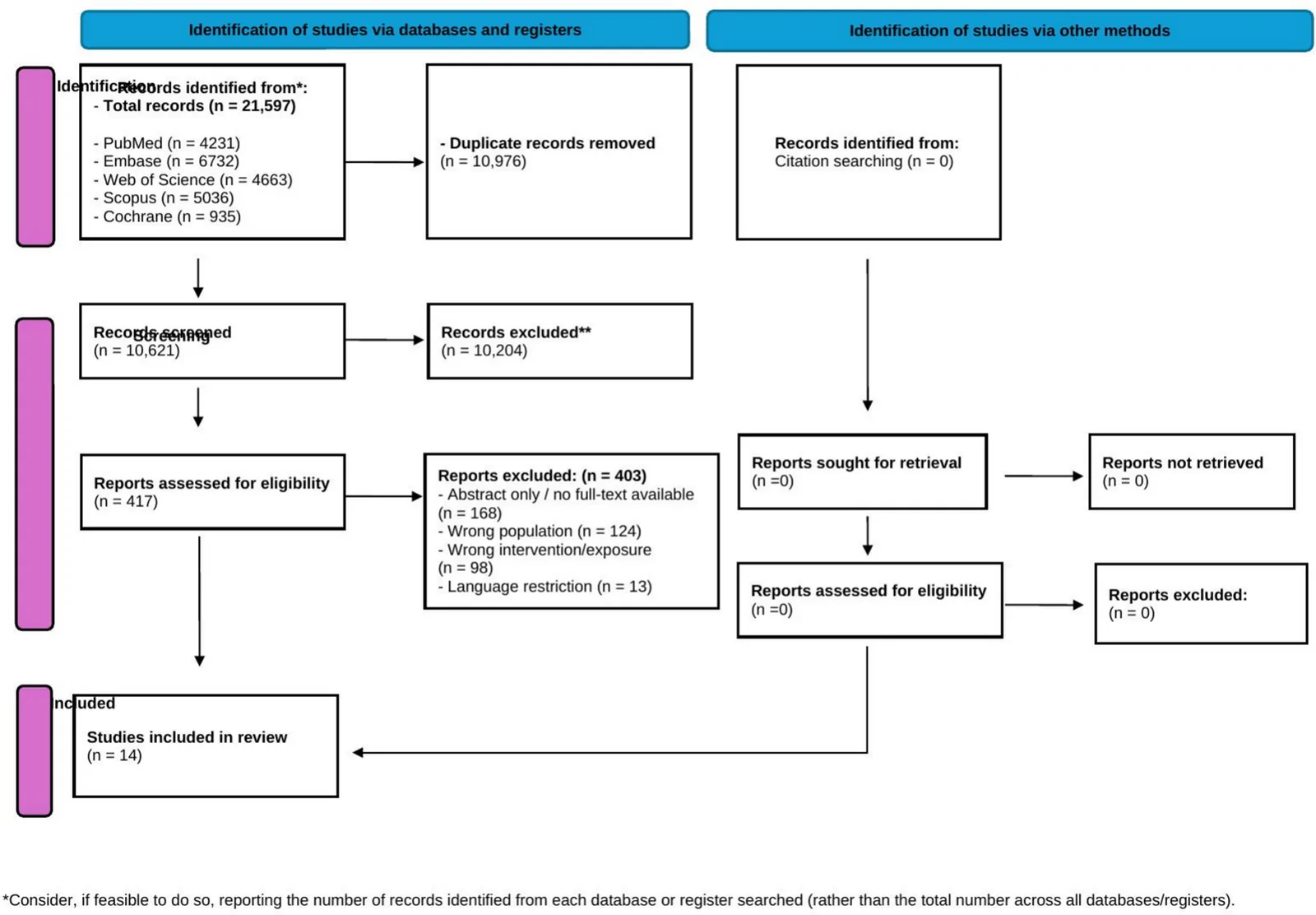
Prisma flowchart

The database search identified 21,597 citations. After duplicate removal, 10,621 unique records underwent title and abstract screening, of which 417 were selected for full-text review. Fourteen studies met all inclusion criteria and were included in this network meta-analysis (PRISMA flow diagram shown in Fig. 1).

### Studies characteristics

The fourteen included studies were published between 2017 and 2025 [18–31]. Most were conducted in the United States, with one Swiss study; thus, the cohort predominantly represented North American populations. Among 9,348 included patients, 200 were enrolled in 2 randomized trials, both comparing eTEP versus rIPOM, whereas the remaining 9,148 patients were derived from retrospective comparative cohorts.

Age and BMI were broadly similar across many cohorts; however, clinically important differences were present in hernia morphology and procedural complexity across approaches. Mean age ranged from the 40 s to early 60 s across cohorts, consistent with the typical hernia repair population. Similarly, body mass index (BMI) values were predominantly in the overweight-to-obese range across studies, suggesting comparability in metabolic risk profiles. These similarities strengthen cross-study comparability regarding postoperative outcomes. Specific baseline characteristics and demography features were summarized across all the mentioned studies (Table 1).

**Table 1:** Baseline and Hernia Characteristics of Included Studies

Study Information					Patient Characteristics				Hernia Morphology					Hernia Location		Hernia Etiology				
Study ID	Arm	Design	Year	Setting	*N*	Age (Mean ± SD)	Female *n* (%)	BMI (Mean ± SD)	Defect Width (cm)	Defect Length (cm)	Mesh Size (cm²)	Overlap (cm)	Defect Area (cm²)	Midline *n* (%)	Lateral *n* (%)	Umbilical *n* (%)	Incisional *n* (%)	Epigastric *n* (%)	Ventral *n* (%)	Other / NR
Kudsi et al. [27] (TA-RM vs TEP/RM)	TA-RM	Retro. cohort	2020	Elective	82	57.8 ± 13.1	60 (54.1%)	32.6 ± 6.5	—	—	393.3 ± 301.9	6.83 ± 2.26	34.5 ± 46.2	—	—	—	54 (65.9%)	—	28 (34.1%)	—
	TEP/RM				82	56.6 ± 14.7	46 (44.7%)	31.3 ± 6.7	—	—	375 ± 282.9	6.5 ± 1.5	35.6 ± 47.5	—	—	—	50 (61%)	—	32 (39%)	—
Kudsi et al. [28]	IPOM	Retro. cohort	2021	Elective	90	47.9 ± 14.5	48 (53.3%)	31.3 ± 6.3	—	—	96.5 ± 37.2	4 ± 1.1	4.1 ± 2.3	—	—	83 (92.2%)	—	9 (10%)	—	—
	TAPP				108	50.2 ± 14.2	30 (27.8%)	30.5 ± 5.7	—	—	117.9 ± 85.2	4.3 ± 1.1	4.4 ± 2.9	—	—	104 (96.3%)	—	12 (11.1%)	—	—
	RM				71	52 ± 13.7	26 (36.6%)	30 ± 6.4	—	—	250 ± 56.8	5.7 ± 0.4	13.5 ± 8.5	—	—	70 (98.6%)	—	14 (19.7%)	—	—
Kudsi et al. [29]	IPOM	Retro. cohort	2022	Elective	197	54.3 ± 14.4	109 (55.3%)	32.6 ± 6.7	2.5 ± 1.1	—	108.9 ± 64.5	4.2 ± 1.1	6.9 ± 7.1	191 (97%)	6 (3%)	—	—	—	108 (54.8%)	—
	TAPP				156	52.1 ± 14.3	53 (34%)	31.9 ± 6.2	2.3 ± 0.75	—	118.9 ± 87.1	4.3 ± 1.1	4.5 ± 3.2	141 (90.4%)	13 (8.3%)	—	—	—	117 (75%)	—
	RS				153	54.6 ± 14.2	52 (34%)	31.1 ± 6.5	3.7 ± 0.75	—	250 ± 56.1	5.6 ± 0.15	9.8 ± 7.5	153 (100%)	0 (0%)	—	—	—	108 (70.6%)	—
	TAR				138	60.2 ± 13.2	82 (59.4%)	33.1 ± 7	7.3 ± 6	—	600 ± 449.5	6.7 ± 3	69.5 ± 91.5	102 (73.9%)	19 (13.8%)	—	—	—	15 (10.9%)	—
Kudsi et al. [30] (lateral incisional)	IPOM	Retro. cohort	2020	Elective	5	58.2 ± 6.7	3 (60%)	34.9 ± 2.2	—	—	207.3 ± 284.4	5.2 ± 1.5	25.3 ± 49.8	—	—	—	—	—	—	—
	TAPP				8	57 ± 13.7	5 (62.5%)	28.9 ± 5	—	—	189.5 ± 105.9	3.9 ± 2.1	11.7 ± 16.8	—	—	—	—	—	—	—
	RM				13	62.1 ± 15.8	10 (76.9%)	30.5 ± 6	—	—	325 ± 186.9	5.8 ± 0.8	15.7 ± 5.2	—	—	—	—	—	—	—
Kudsi et al. [31] (IPOM vs TEP-RM)	IPOM	Retro. cohort	2020	Elective	68	57.8 ± 12.7	34 (50%)	31.2 ± 5.7	—	—	134.2 ± 48.3	4.2 ± 1.1	13.6 ± 15.5	64 (94.1%)	4 (5.9%)	—	40 (58.8%)	—	28 (41.2%)	—
	TEP-RM				68	56.5 ± 15.9	32 (47.1%)	31.6 ± 7	—	—	300 ± 113.6	5.7 ± 0.4	16.97 ± 8.9	59 (86.8%)	9 (13.2%)	—	28 (41.2%)	—	40 (58.8%)	—
Vargas et al. [18]	IPOM	Retro. cohort	2022	Elective	4486	56.8 ± 15.28	2235 (49.8%)	32.33 ± 6.67	3.0 ± 1.5	3.7 ± 3	131.4 ± 64.1	—	—	4208 (93.9%)	413 (9.2%)	1284 (28.6%)	2832 (63.2%)	393 (8.8%)	—	—
	PP				1829	54 ± 16.32	781 (42.7%)	31.70 ± 6.23	2.3 ± 0.7	3.0 ± 1.5	114.1 ± 53.6	—	—	1581 (86.5%)	283 (15.5%)	873 (47.7%)	680 (37.2%)	226 (12.4%)	—	—
	RM				948	56.33 ± 14.85	496 (52.3%)	31.90 ± 6.24	4.3 ± 1.5	7 ± 5.2	311.6 ± 169.1	—	—	902 (95.2%)	100 (10.6%)	187 (19.7%)	690 (72.8%)	103 (10.9%)	—	—
Gokcal et al. [19]	IPOM	Retro. cohort	2019	Elective	104	50.9 ± 14.5	44 (42.3%)	31.22 ± 5.60	2.3 ± 0.75	2.3 ± 0.75	96.6 ± 37.2	4.2 ± 1.1	4.5 ± 2.95	99 (95.2%)	5 (4.8%)	—	29 (27.9%)	—	75 (72.1%)	—
	TAPP				104	51.2 ± 14.1	41 (39.4%)	31.60 ± 5.22	2.3 ± 0.75	2.5 ± 0.75	118.9 ± 87.5	4.3 ± 1.1	4.6 ± 2.95	99 (94.2%)	6 (5.8%)	—	27 (26%)	—	77 (74%)	—
Kennedy et al. [20]	IPOM	Retro. cohort	2017	Elective	27	45.6 ± 13.1	22 (81.4%)	31.8 ± 9.6	—	—	—	—	3.40 ± 1.51	—	—	—	—	—	—	—
	TAPP				36	44.3 ± 13.3	24 (66.7%)	31.0 ± 6.4	—	—	—	—	3.98 ± 2.61	—	—	—	—	—	—	—
Pacheco et al. [21]	TARM	Retro. cohort	2024	NR	36	57.47 ± 12.0	18 (50%)	33.24 ± 6.5	5.10 ± 1.4	—	329.06 ± 70.3	—	—	—	—	—	20 (55.5%)	—	16 (44.4%)	—
	ETEP				60	57.35 ± 11.2	32 (53.3%)	30.47 ± 4.7	4.9 ± 2.6	—	724.56 ± 193.7	—	—	—	—	—	32 (53.3%)	—	28 (46.6%)	—
Petro et al. [23] (REVEAL 1-year)	ETEP	RCT	2023	Elective	51	51.90 ± 16.25	22 (45%)	33.76 ± 5.15	4 ± 1.5	6 ± 5.3	360.3 ± 121.3	—	—	—	—	—	—	—	—	—
	IPOM				49	57.23 ± 15.50	25 (49%)	30.86 ± 7.36	3.50 ± 2.3	4.8 ± 4.8	200 ± 57.3	—	—	—	—	—	—	—	—	—
Pini et al. [24]	ETEP	Retro. cohort	2024	NR	32	57.8 ± 11.9	5 (15.6%)	27.1 ± 5.5	—	—	214 ± 42	—	2.5 ± 0.9	—	—	27 (84.4%)	7 (21.9%)	2 (6.2%)	—	—
	ETAPP				21	60.9 ± 16.6	3 (14.2%)	29.5 ± 6.3	—	—	211 ± 245	—	2.5 ± 0.8	—	—	16 (76.2%)	2 (9.5%)	2 (9.5%)	—	—
Saleh et al. [25]	IPOM	Retro. cohort	2025	NR	44	57.4 ± 14.6	22 (50.0%)	36.0 ± 9.4	—	—	—	34.7 ± 51.4	—	—	—	—	—	—	—	—
	ETEP				44	56.8 ± 13.7	18 (40.9%)	34.2 ± 7.7	—	—	—	40.7 ± 41.4	—	—	—	—	—	—	—	—
Zaman et al. [26]	ETEP	Retro. cohort	2023	Elective	50	53.5 ± 13.6	31 (62%)	33.1 ± 7.8	—	—	432.8 ± 246.4	109.1 ± 148.5	—	—	—	—	45 (90%)	—	5 (10%)	—
	TA-SM				108	51.0 ± 12.7	67 (62%)	33.7 ± 7.7	—	—	137.9 ± 146.8	31.8 ± 59.3	—	—	—	—	98 (90.7%)	—	10 (9.26%)	—
Petro et al. [22] (REVEAL 30-day)	IPOM	RCT	2022	Elective	49	57.23 ± 15.50	22 (45%)	30.86 ± 7.36	3.50 ± 2.3	4.8 ± 4.8	200 ± 57.3	—	—	—	—	—	—	—	—	—
	ETEP				51	52.90 ± 16.25	25 (49%)	33.76 ± 5.15	4 ± 1.5	6 ± 5.3	360.3 ± 121.3	—	—	—	—	—	—	—	—	—

*Retro*. retrospective; *RCT* randomised controlled trial; *NR* not reported; *NA* not available; *TA-RM* transabdominal retromuscular; *TEP/RM* totally extraperitoneal retromuscular; *IPOM* intraperitoneal onlay mesh; *TAPP* transabdominal preperitoneal; *RS* robotic surgery; *TAR* transversus abdominis release; *RM* retromuscular; *PP* prepertional; *TARM* transabdominal retromuscular; *ETEP* enhanced view totally extraperitoneal; *ETAPP* enhanced view transabdominal preperitoneal; *TA-SM* transabdominal sublay mesh; *TEP-RM* totally extraperitoneal retromuscular. Values presented as mean ± SD unless otherwise stated. — = not reported

### Procedural characteristics

The range of surgical techniques studied includes a variety of Robotic methods for hernia repair, indicating the changing trends in practice. The type of surgical technique compared in the studies was rIPOM, rTAPP, rTEP-RM, rTAR, rRM, rTA-RM, rRS, PP (preperitoneal group), RM (retromuscular group), eTEP, eTAPP, TARM, and TA-SM. This heterogeneity of surgical techniques enabled extensive study and comparison of various technical procedures and end results. Detailed procedural characteristics, including defect size, mesh dimensions, overlap distance, and anatomic location. Hernia types across studies were highly heterogeneous, encompassing midline, lateral, umbilical, incisional, epigastric, and ventral defects. Incisional and ventral hernias predominated in study populations, consistent with their prevalence in routine surgical practice.

#### Defect measurements

Reported defect width ranged from approximately 2.3 to 7.3 cm across studies, with larger defects more frequently represented in TAR and retromuscular reconstruction cohorts than in IPOM or TAPP cohorts. Hernia types were also heterogeneous and included umbilical, incisional, epigastric, midline, lateral, and mixed ventral defects. These differences are clinically relevant and may have influenced both treatment selection and observed outcomes. Larger defects (> 5 cm) generally require extended reconstruction techniques. Mesh dimensions similarly varied based on surgical technique and defect characteristics, ranging from 600 to 1200 cm^2^ (specified from Table 2 exact range). Mesh overlap distance ranged from 4 to 7 cm across studies, consistent with standard surgical principles emphasizing adequate mesh coverage beyond defect margins to minimize recurrence.

#### Hernia location and etiology

Hernia location and etiology varied across studies. Midline hernias predominated, consistent with their higher prevalence in abdominal wall pathology; however, lateral, incisional, umbilical, and epigastric hernias were also frequently represented. Incisional hernias (complicating prior surgical interventions) were present in > 50% of cases in several studies, highlighting their clinical significance. The study populations also included primary and recurrent ventral and umbilical hernias, reflecting real-world surgical practice.

### Risk of bias assessment fr Q7 it refer to ref 17 

Two independent assessors evaluated risk of bias using domain-specific tools. Non-randomized studies (*n* = 12) were assessed with ROBINS-I, demonstrating moderate-to-serious risk of bias primarily due to confounding and selection bias (Supplementary Fig. 1). The two randomized trials [22] were assessed with Cochrane RoB 2, showing low risk of bias (Supplementary Fig. 2).

### Operative outcomes

#### Console time (minutes)

Six studies, including 1,241 patients and seven hernia repair techniques were analyzed using a random-effects model. rTAR (MD = 141.11, 95% CI: 118.65 to 163.57; *p* < 0.001), rRM (MD = 63.23, 95% CI: 13.06 to 113.40; *p* = 0.014), rTA-RM (MD = 53.17, 95% CI: 12.90 to 93.44; *p* = 0.010), and rRS (MD = 25.94, 95% CI: 9.45 to 42.43; *p* = 0.002) demonstrated significantly longer console times compared with rIPOM. However, rTEP-RM and rTAPP showed no significant differences. According to SUCRA, rIPOM ranked highest probability of achieving shorter console time (92%), followed by rTAPP (87%), and rTAR ranked lowest (0%). There was substantial heterogeneity within designs (I^2^ = 78.8%, *p* = 0.050). (Supplementary Figs. 3, 4).

#### Total operative time (minutes)

Thirteen studies including 2,105 patients and eleven hernia repair techniques were analyzed using a common-effects model. Compared with rIPOM, several approaches were associated with significantly longer operative times, including rTAR (MD = 148.11, 95% CI: 129.58 to 166.65; *p* < 0.001), eTAPP (MD = 73.31, 95% CI: 36.07 to 110.56; *p* < 0.001), eTEP (MD = 53.31, 95% CI: 40.52 to 66.11; *p* < 0.001), TARM (MD = 53.14, 95% CI: 32.20 to 74.09; *p* < 0.001), TA-SM (MD = 50.81, 95% CI: 20.06 to 81.56; *p* = 0.001), rTA-RM (MD = 50.50, 95% CI: 18.26 to 82.74; *p* = 0.002), rRM (MD = 35.05, 95% CI: 24.39 to 45.71; *p* < 0.001), rRS (MD = 28.28, 95% CI: 20.18 to 36.39; *p* < 0.001), and rTEP-RM (MD = 16.00, 95% CI: 0.41 to 31.59; *p* = 0.044). In contrast, rTAPP showed no significant difference. SUCRA rankings indicated rTAPP (96%) and rIPOM (94%) had the highest probabilities of shorter operative time, while rTAR ranked the lowest (0%). Heterogeneity within designs was not significant (I^2^ = 53.5%, p = 0.575). (Supplementary Figs. 3, 4).

#### Intraoperative complication rate

Six studies including 8,441 patients and nine hernia repair techniques were analyzed using a common-effects model. No approaches demonstrated significant differences in intraoperative complication rates compared with rIPOM. SUCRA rankings indicated rTEP-RM ranked first (81%), followed by rTAPP (76%), and rTAR ranked last (23%). There was no heterogeneity within designs (I^2^ = 0%). (Supplementary Figs. 4, 5).

### Early postoperative outcomes

#### Postoperative pain score

Nine studies including 8,417 patients and eight hernia repair techniques were analyzed using a common-effects model. Compared with rIPOM, PP (SMD − 0.35, 95% CI − 0.41 to − 0.30; *p* < 0.001) and rRM (SMD − 0.14, 95% CI − 0.21 to − 0.07; *p* < 0.001) were associated with lower postoperative pain scores, whereas rTEP-RM was associated with higher pain scores (SMD 0.35, 95% CI 0.01 to 0.69; *p* = 0.044). Other approaches did not show significant differences. SUCRA ranked PP first (99%), followed by rTAPP (72%), and rTEP-RM ranked last (15%). There was no significant heterogeneity within designs (I^2^ = 86.6%, *p* = 0.495). Figures 4 and 5.

#### Overall complication rate

**Fig. 2:**
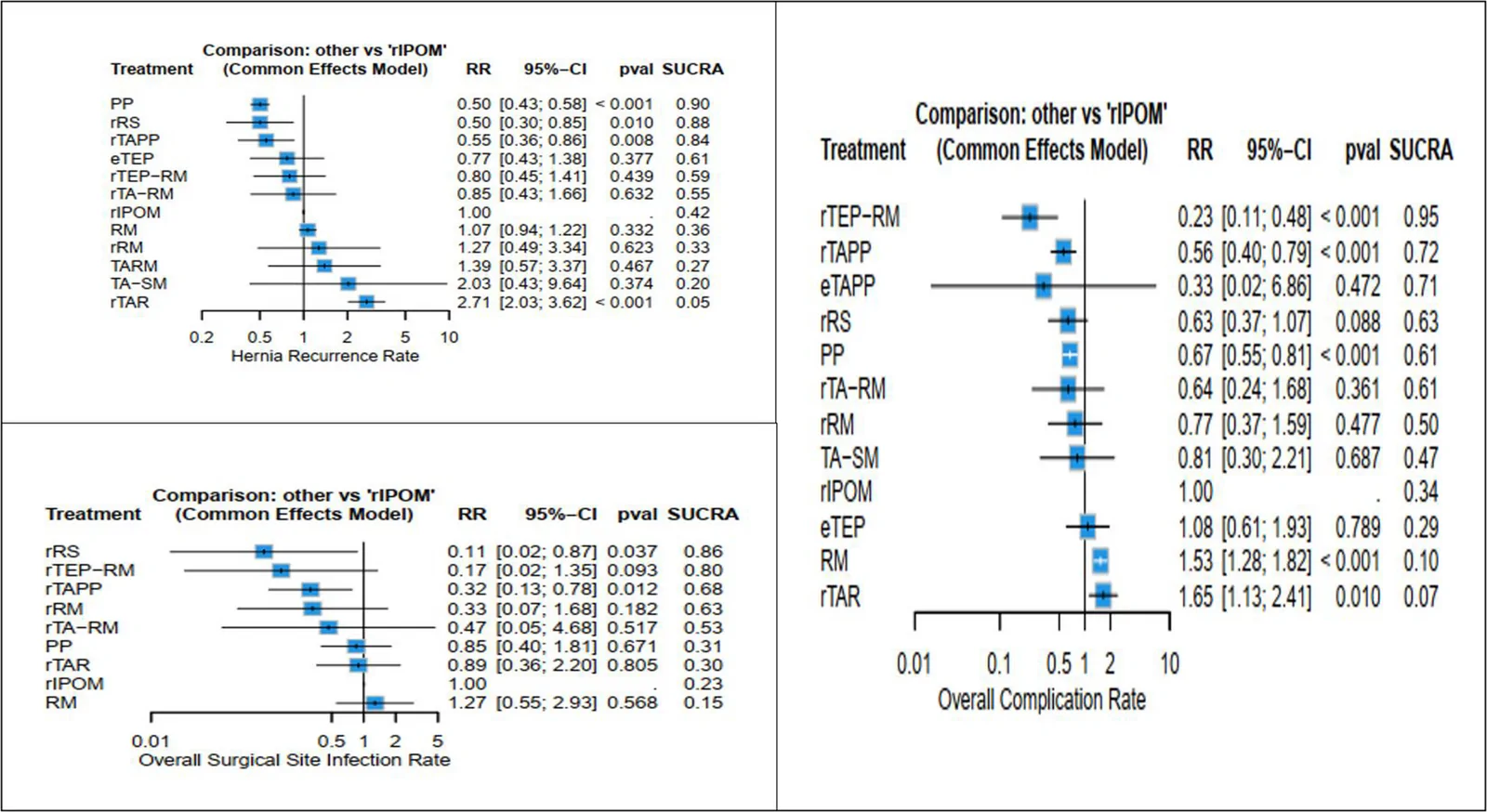
Forest plot meta-analysis for recurrence rate, surgical site infection, and overall complication rate

**Fig. 3:**
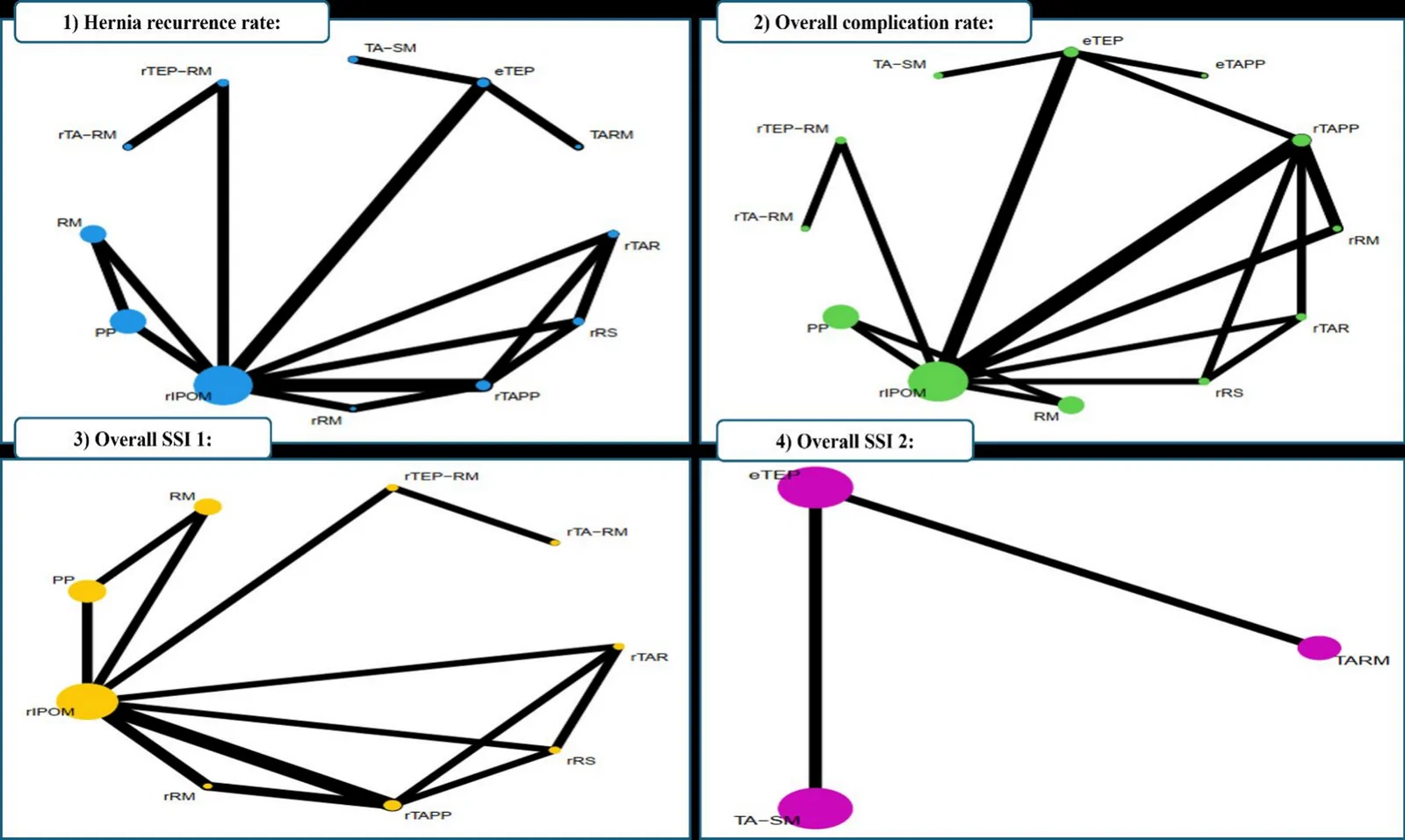
Network plots from the meta-analysis for a specific outcome (e.g., hernia recurrence rate, overall complication rate, and overall surgical site

Fourteen studies encompassing 9,348 patients and twelve hernia repair techniques were analyzed using a random-effects model. The analysis showed that rTEP-RM (RR = 0.23, 95% CI:0.07 to 0.74; *p* = 0.014) and rTAPP (RR = 0.55, 95% CI:0.30 to 0.99; *p* = 0.048) demonstrated significantly lower overall complication rates compared with rIPOM. Other approaches did not show significant differences. According to SUCRA rankings, rTEP-RM ranked first (92%), followed by rTAPP (70%), and rTAR ranked last (19%). There was moderate heterogeneity within designs (I^2^ = 43.5%, *p* = 0.026). Figures 2 and 3

#### Seroma formation rate

**Fig. 4:**
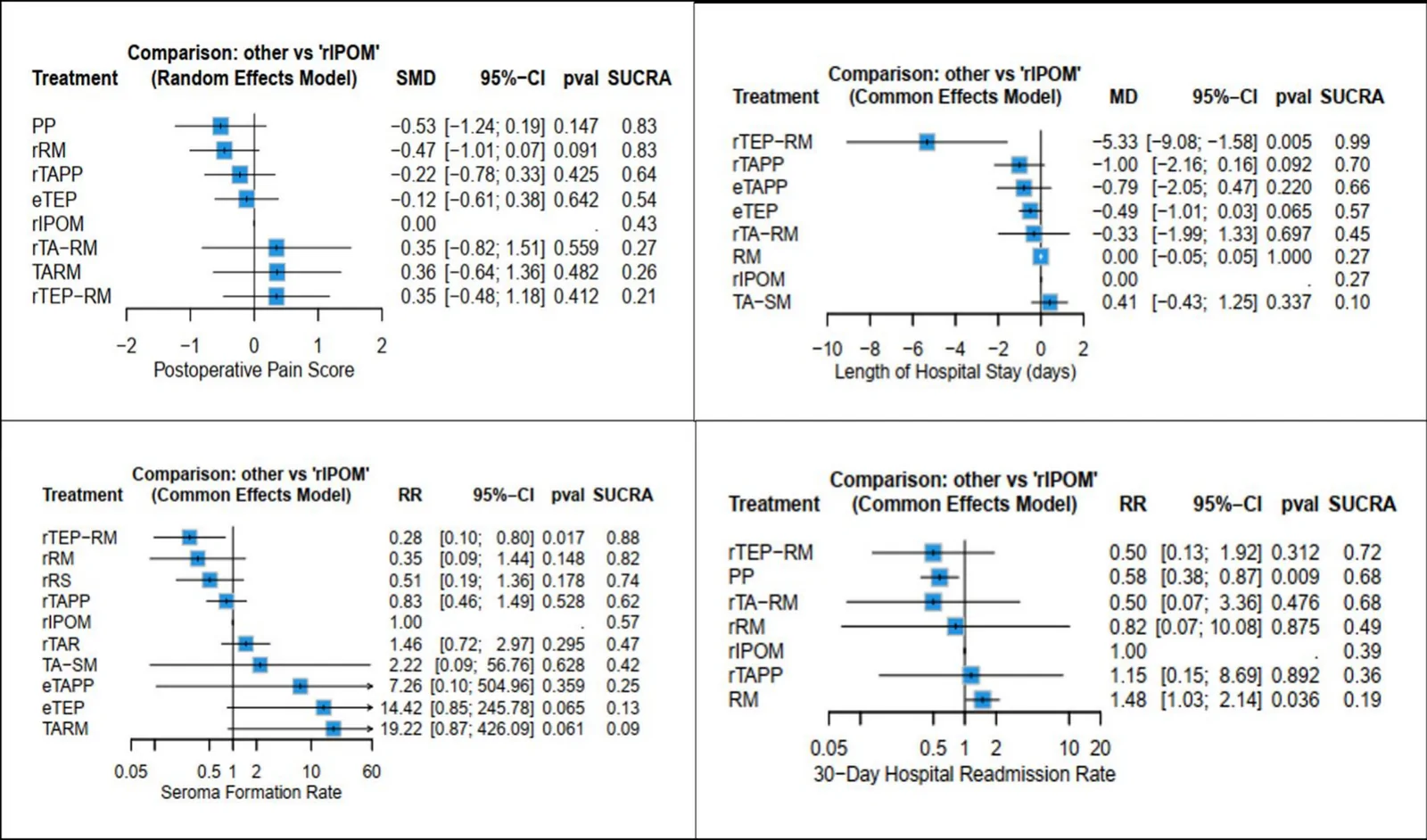
Forest plot meta-analysis for postoperative pain score, length of hospital stay, seroma formation rate and 30-day hospital readmission rate

**Fig. 5:**
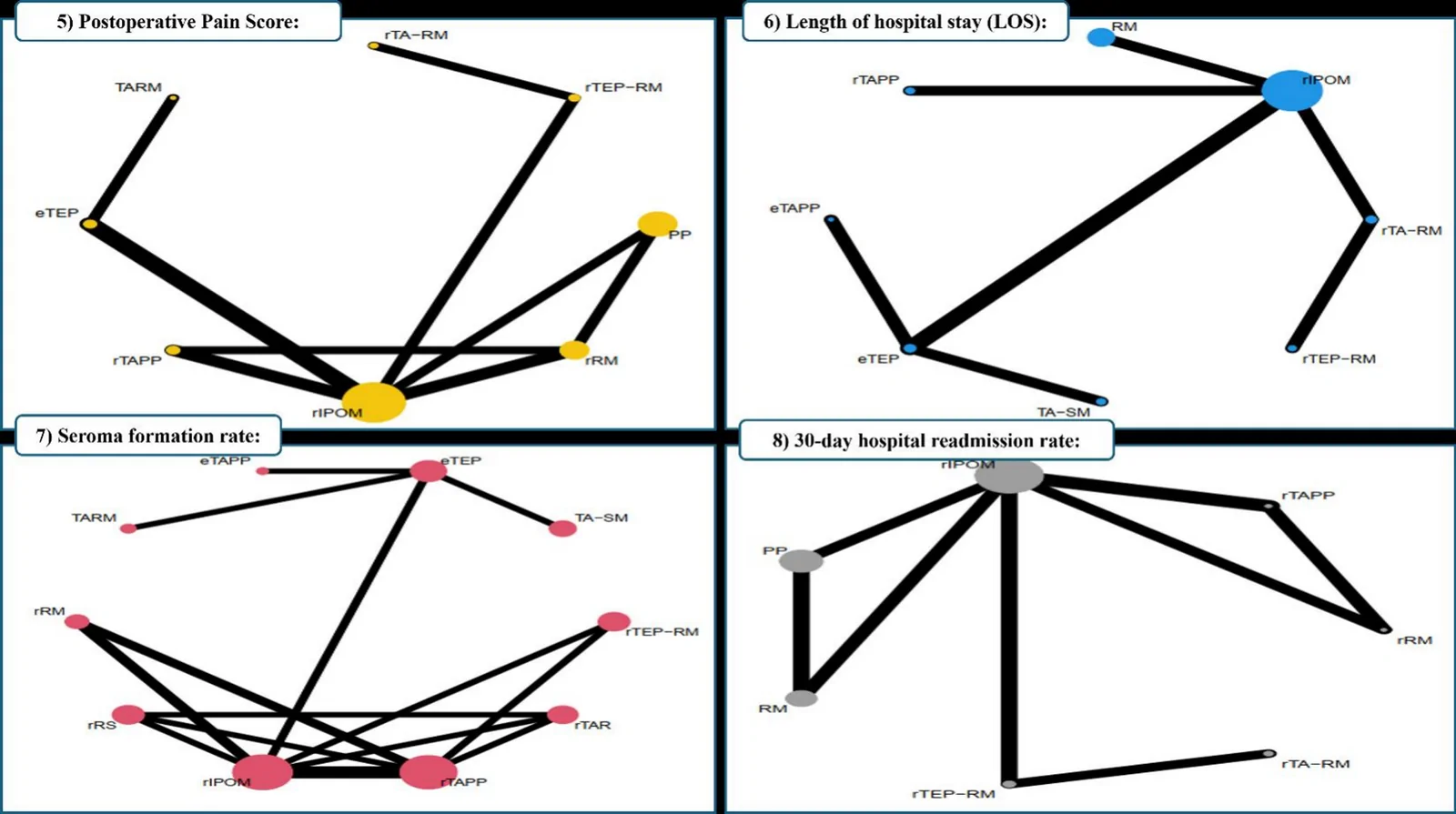
Network plots from the meta-analysis for a specific outcome (e.g., postoperative pain score, length of hospital stay (LOS), seroma formation rate, and 30-day hospital readmission rate)

Eleven studies including 1,897 patients and ten hernia repair techniques were analyzed using a common-effects model. The analysis showed that rTEP-RM (RR = 0.28, 95% CI:0.10 to 0.80; *p* = 0.017) significantly reduced seroma formation compared with rIPOM. Other approaches did not show significant differences. According to SUCRA rankings, rTEP-RM ranked first (88%), followed by rRM (82%), and TARM ranked last (9%). There was no significant heterogeneity within designs (I^2^ = 0%, *p* = 0.365). Figures 4 and 5.

#### Hematoma rate

Seven studies including 1,490 patients and seven hernia repair techniques (rTA − RM, rTEP − RM, rTAPP, rRS, rTAR, rRM, rIPOM) were analyzed using a common-effects model. No approaches demonstrated significant differences in hematoma rates compared with rIPOM. There was no heterogeneity within designs (I^2^ = 0%, *p* = 0.664). rTA-RM ranked first (78%), followed by rTEP-RM (66%), and rIPOM ranked last (18%), according to SUCRA rankings. A subnetwork analysis was performed for the same outcome in two studies including 254 patients and three hernia repair techniques (eTEP, TA-SM, TARM). None of the approaches demonstrated significant differences compared with eTEP. According to SUCRA rankings, eTEP ranked first (69%), followed by TARM (47%), and TA-SM ranked last (33%) (Supplementary Figs. 5, 6).

#### Overall surgical site infection rate

Seven studies including 8,690 patients and nine hernia repair techniques (rRS, rTEP-RM, rTAPP, rRM, rTA-RM, PP, rTAR, rIPOM, RM) were analyzed using a common-effects model. The analysis showed that rRS (RR = 0.11, 95% CI:0.02 to 0.87; *p* = 0.037) and rTAPP (RR = 0.32, 95% CI:0.13 to 0.78; *p* = 0.012) demonstrated significantly lower overall surgical site infection rates compared with rIPOM. Other approaches did not show significant differences. According to SUCRA rankings, rRS ranked first (86%), followed by rTEP-RM (80%), and RM ranked last (15%). There was no significant heterogeneity within designs (I^2^ = 0%, *p* = 0.839). Two studies, including 254 patients and three hernia repair techniques (eTEP, TA-SM, TARM) were analyzed as a subnetwork for surgical site infection rates using a common-effects model. None of the approaches demonstrated significant differences compared with eTEP. According to SUCRA rankings, eTEP ranked first (73%), followed by TA-SM (55%), and TARM ranked last (22%). Figure 2 and 3.

#### Length of hospital stay

Eight studies including 6,341 patients and eight hernia repair techniques were analyzed using a common-effects model. Compared with rIPOM, only rTEP-RM was associated with a significantly shorter hospital stay (MD = − 5.33, 95% CI: − 9.08 to − 1.58; *p* = 0.005). Other approaches did not demonstrate significant differences compared with rIPOM. According to SUCRA rankings, rTEP-RM (99%) had the highest probability of achieving shorter hospital stay, followed by rTAPP (70%), while TA-SM ranked the lowest (10%). There was no evidence of heterogeneity (I^2^ = 0%, *p* = 0.679). Figures 4 and 5.

### Late outcomes

#### 30-Day hospital readmission rate

Five studies including 7,652 patients and seven hernia repair techniques were analyzed using a common-effects model. The analysis showed that PP (RR = 0.58, 95% CI:0.38 to 0.87; *p* = 0.009) was associated with a significantly lower 30-day hospital readmission rate than rIPOM, while RM (RR = 1.48, 95% CI:1.03 to 2.14; *p* = 0.036) was associated with a significantly higher readmission rate. Other approaches did not show significant differences. According to SUCRA rankings, rTEP-RM ranked first (72%), followed by PP (68%), and RM ranked last (19%). There was no heterogeneity within designs. Figures 4 and 5.

#### Reoperation rate

Six studies including 7,785 patients and nine hernia repair techniques were analyzed using a common-effects model. RM (RR = 2.60, 95% CI:1.50 to 4.49; *p* < 0.001) demonstrated a significantly higher reoperation rate compared with rIPOM, while other approaches showed no significant differences. According to SUCRA rankings, PP ranked first (80%), followed by rTEP-RM (77%), and TARM ranked last (17%). No heterogeneity was observed within designs. (Supplementary Figs. 3, 4).

#### Hernia recurrence rate

Ten studies, including 8,867 patients and twelve techniques were analyzed using a common-effects model. PP (RR = 0.50, 95% CI:0.43 to 0.58; *p* < 0.001), rRS (RR = 0.50, 95% CI:0.30 to 0.85; *p* = 0.010), and rTAPP (RR = 0.55, 95% CI:0.36 to 0.86; *p* = 0.008) significantly reduced recurrence rates compared with rIPOM, whereas rTAR showed a significantly higher recurrence rate (RR = 2.71, 95% CI:2.03 to 3.62; *p* < 0.001). Other approaches showed no significant differences. SUCRA rankings placed PP first (90%), followed by rRS (88%), and rTAR last (5%). No significant heterogeneity was observed within designs (I^2^ = 12.1%, *p* = 0.999). Because recurrence follow-up duration, completeness, and ascertainment methods varied substantially across studies, the recurrence network should be interpreted cautiously. The observed estimates reflect short- to mid-term comparative signals rather than definitive long-term comparative effectiveness. Figures 2 and 3.

### Summary of findings and certainty of evidence

Certainty of evidence was assessed using CINeMA. Overall, evidence was low for most comparisons and very low for a few, with no moderate- or high-certainty estimates. Downgrading was mainly driven by risk of bias from predominantly retrospective studies and by imprecision due to wide confidence intervals and limited contributing studies. Sparse data and possible reporting bias were additional concerns. No major inconsistency was identified between direct and indirect evidence.

For hernia recurrence, PP was associated with lower recurrence than rIPOM (RR 0.50, 95% CI 0.43–0.58; low certainty), whereas rTAR was associated with higher recurrence risk (RR 2.71, 95% CI 2.03–3.62; low certainty). For overall complications, rTEP-RM (RR 0.23, 95% CI 0.07–0.74) and rTAPP (RR 0.55, 95% CI 0.30–0.99) favored rIPOM, but certainty was low. For surgical site infection, rRS (RR 0.11, 95% CI 0.02–0.87) and rTAPP (RR 0.32, 95% CI 0.13–0.78) were associated with lower event rates than rIPOM, again with low certainty. PP was also associated with lower 30-day readmission (RR 0.58, 95% CI 0.38–0.87; low certainty), whereas RM was associated with higher readmission (RR 1.48, 95% CI 1.03–2.14; low certainty). Given the overall low certainty, these findings should be considered hypothesis-generating. Detailed CINeMA judgments are provided Table 2 and Supplementary Table 2.

**Table 2:** Summary of main network meta-analysis findings comparing alternative robotic techniques with rIPOM

Outcome & comparison vs rIPOM	Relative effect (95% CI)*	Absolute effect per 1,000 patients†	Certainty of evidence (GRADE)	Key reasons for downgrading‡
HERNIA RECURRENCE, 1 YEAR				
PP vs rIPOM	RR 0.50 (0.43–0.58)	112 fewer events (127 fewer to 94 fewer)	⨁⨁◯◯ LOW	Downgraded 2 levels for risk of bias (network dominated by retrospective cohorts) and 1 level for imprecision (limited number of studies and events)
rTAPP vs rIPOM	RR 0.55 (0.36–0.86)	101 fewer events (143 fewer to 31 fewer)	⨁⨁◯◯ LOW	Risk of bias (2 levels); imprecision (1 level, confidence interval includes small and moderate benefit)
rRS vs rIPOM	RR 0.50 (0.30–0.85)	112 fewer events (156 fewer to 34 fewer)	⨁⨁◯◯ LOW	Risk of bias (2); imprecision (1, relatively few events)
rTAR vs rIPOM	RR 2.71 (2.03–3.62)	382 more events (230 more to 585 more)	⨁⨁◯◯ LOW	Risk of bias (2); potential confounding by indication (technique used for more complex hernias)
Other techniques vs rIPOM	Effect estimates close to or crossing no difference; wide CIs	Direction and magnitude uncertain	⨁◯◯◯ VERY LOW	Risk of bias (2); very serious imprecision and possible reporting bias
OVERALL COMPLICATIONS, 30 DAYS				
rTEP-RM vs rIPOM	RR 0.23 (0.07–0.74)	122 fewer events (147 fewer to 41 fewer)	⨁⨁◯◯ LOW	Risk of bias (2); imprecision (1, few studies and events)
rTAPP vs rIPOM	RR 0.55 (0.30–0.99)	71 fewer events (111 fewer to 2 fewer)	⨁⨁◯◯ LOW	Risk of bias (2); imprecision (1, confidence interval includes little or no benefit)
Other techniques vs rIPOM	No important difference; wide CIs	Effect uncertain	⨁◯◯◯ VERY LOW	Risk of bias (2); serious imprecision
OVERALL SURGICAL-SITE INFECTION, 30 DAYS				
rRS vs rIPOM	RR 0.11 (0.02–0.87)	44 fewer events (48 fewer to 6 fewer)	⨁⨁◯◯ LOW	Risk of bias (2); imprecision (1, very few events, wide confidence interval)
rTAPP vs rIPOM	RR 0.32 (0.12–0.86)	34 fewer events (43 fewer to 7 fewer)	⨁⨁◯◯ LOW	Risk of bias (2); imprecision (1, few events)
Other techniques vs rIPOM	No consistent differences; wide CIs	Effect uncertain	⨁◯◯◯ VERY LOW	Risk of bias (2); imprecision; sparse data
REOPERATION, 30 DAYS				
PP vs rIPOM	RR 0.45 (0.22–0.91)	8 fewer events (11 fewer to 1 fewer)	⨁⨁◯◯ LOW	Risk of bias (2); imprecision (1, few events)
Other techniques vs rIPOM	No consistent differences; CIs cross 1	Effect uncertain	⨁◯◯◯ VERY LOW	Risk of bias (2); serious imprecision
SEROMA, 30 DAYS				
rTEP-RM vs rIPOM	RR 0.28 (0.10–0.80)	40 fewer events (50 fewer to 11 fewer)	⨁⨁◯◯ LOW	Risk of bias (2); imprecision (1, few events)
HEMATOMA, 30 DAYS				
All techniques vs rIPOM	No consistent differences; very few events	Effect highly uncertain	⨁◯◯◯ VERY LOW	Risk of bias (2); very serious imprecision
INTRAOPERATIVE COMPLICATIONS				
All techniques vs rIPOM	No important differences; CIs generally include no effect	Effect uncertain	⨁◯◯◯ VERY LOW	Risk of bias (2); serious imprecision; events rare
OPERATIVE AND CONSOLE TIME				
rTAPP vs rIPOM	No important difference in total operative time; console time similar	Not applicable	⨁⨁◯◯ LOW	Risk of bias (2); some inconsistency; judgment that small time differences are unlikely to be clinically important
Most other techniques vs rIPOM (eTEP, eTAPP, TA-SM, RM, rRM, rTEP-RM, rTA-RM, rTAR)	Generally longer operative and/or console time compared with rIPOM	Not applicable	⨁⨁◯◯ LOW	Risk of bias (2); imprecision not serious (narrow confidence intervals); effects consistent in direction

^*^ Relative risks (RR) are from the network meta-analysis. Confidence intervals are 95% intervals. ‘Other techniques’ denotes comparisons with sparse data, highly imprecise estimates, or very low certainty. † Absolute effects for binary outcomes were calculated using the median baseline risk observed in rIPOM arms across included studies for each outcome. Median baseline risks used were: hernia recurrence 223/1,000; overall complications 159/1,000; SSI 49/1,000; reoperation 14/1,000; and seroma 56/1,000. For techniques with sparse data, highly imprecise estimates, or substantial between-study heterogeneity, absolute effects were not calculated to avoid conveying misleading precision. ‡ Certainty was downgraded per GRADE domains: risk of bias, inconsistency, indirectness, imprecision, and publication bias

## Discussion

To our knowledge, this is the most comprehensive network meta-analysis of robotic ventral hernia repair techniques to date, synthesizing 14 studies (2017–2025) of 9,348 patients comparing 12 distinct configurations. Robotic assistance facilitates precise mesh placement in the preperitoneal and retro rectus planes with reduced operative difficulty [32]. The historical shift from open to laparoscopic techniques proved transformative; the current transition to robotic approaches appears similarly promising. This network meta-analysis enables both direct and indirect comparisons across the entire intervention landscape.

Our findings suggest that (1) rIPOM and rTAPP were associated with shorter operative times than several alternative techniques within the current evidence network; however, these rankings should be interpreted cautiously because most comparisons were informed by observational data and low-certainty evidence (SUCRA rankings: 94% and 96%, respectively), while rTAR required the longest operative duration and ranked last (SUCRA: 0%) These longer operative times likely reflect procedural complexity and case selection rather than inefficiency per se, particularly for techniques that are preferentially used for larger or more complex hernias (for example, rTAR and retro muscular reconstructions); (2) intraoperative complications and hematoma occurrence were similar among all interventions, whereas only rTEP-RM and rTAPP demonstrated significantly lower overall complications than rIPOM, in line with the SUCRA ranking; (3) our analysis reveals both a significant reduction in seroma formation and hospital length of stay and an increase in postoperative pain scores by rTEP-RM in comparison to rIPOM, whereas PP and rRM reduced postoperative pain in contrast with rIPOM; (4) Pooled preperitoneal-plane approaches were associated with lower short-term recurrence and readmission estimates than rIPOM; however, these signals may partly reflect selection of less complex hernias and should not be interpreted as definitive superiority. Likewise, the less favorable recurrence and complication estimates observed for rTAR may partly reflect confounding by indication, because TAR is commonly reserved for larger and more complex defects. Accordingly, the current network should be viewed primarily as a map of available comparative evidence and as a hypothesis-generating basis for future prospective trials rather than a definitive ranking of robotic ventral hernia techniques.

The rTEP-RM technique was associated with lower overall complication rates, reduced seroma formation, and shorter hospital stay compared with rIPOM; however, these findings are supported by low-certainty evidence and should be interpreted cautiously. However, the retro muscular variant of the totally extraperitoneal robotic technique was the only intervention to increase postoperative pain in relation to rIPOM. The higher postoperative pain estimate observed with rTEP-RM relative to rIPOM should be interpreted cautiously. Although an anatomical explanation is plausible, the available evidence is insufficient to determine whether this reflects the procedure itself, differences in case selection, or variation in postoperative analgesic protocols[33]. Other techniques either place the mesh in a different location (pre-peritoneal or intraperitoneal), or from a different perspective such as the trans-abdominal entry which often involves the risk of small bowel injury and higher requirements of adhesion lysis [31]. Moreover, performing rTEP-RM involves the potential risk of the development of interparietal hernias and must be meticulously manipulated to avoid wound dehiscence by executing a transversus abdominis release (TAR) when deemed necessary [27].

Nevertheless, in this network meta-analysis, rTAR was associated with the longest console and operative times when compared with rIPOM, and SUCRA-assigned values placed rTAR last for intraoperative complications, overall complications, and hernia recurrence. These estimates should not be interpreted as evidence of true inferiority, because rTAR is reserved for substantially larger and more complex defects than the other approaches in the network (mean defect width [7.3] cm and mesh area [600] cm^2^ in the rTAR arms vs [2.5] cm and [109] cm^2^ in the rIPOM arms; Supplementary Table 1) and is therefore strongly susceptible to confounding by indication. In an exploratory analysis excluding rTAR from the network, the comparative estimates among the intraperitoneal and preperitoneal approaches were not materially changed, supporting the robustness of the principal findings. rTAR remains the preferred technique for large and complex ventral hernia repair [34]; its inclusion here serves to map the comparative evidence landscape rather than to position it as a competing first-line option, and its results should be interpreted within a patient-centered, defect-stratified framework of surgical decision-making.

With reference to the intraperitoneal onlay mesh intervention, both the preperitoneal and the retro muscular robotic VHR (PP and rRM) displayed a reduction in postoperative pain scores. The finding that rRM reduces postoperative pain scores in comparison to rIPOM answers previous indications for a comparison between the two robotic techniques [35]. Despite this, only PP reduced readmission and recurrence rates, with a high probability of reducing reoperation, while rRM was confirmed to increase 30-day readmission rates. Robotic eTEP, a PP technique, has the benefit of avoiding flap opening and closure which may clarify the ranking of eTEP as first by SUCRA in the subnetwork meta-analyses for hematoma and surgical site infection rates in comparison to TA-SM and rTARM. Nonetheless, the steep learning curve constitutes a problem that might lead surgeons to adopt other techniques instead [32].

rTAPP showed a favorable early postoperative profile in this network, particularly for operative time, overall complications, and SSI relative to rIPOM. However, these findings should not be interpreted as definitive superiority because the supporting evidence remains predominantly observational and may be influenced by case selection and hernia complexity, and it was ranked second in the SUCRA results for intraoperative complication rates despite the lack of statistically significant differences in the common-effects model. No difference was found between operative times for rTAPP and rIPOM, and rTAPP reduced overall complication rates in comparison with rIPOM, which is in line with previous research and validates the finding of Kudsi et al. [29] that rTAPP had the best 90-day freedom from complication (FFC) rate versus rIPOM, rTAR, and rRS [19, 20, 28, 29]. Furthermore, rTAPP was corroborated to reduce surgical site infection rates vis-à-vis rIPOM, which is consistent with Gokcal et al. [19] and may be due to the generally larger mesh-to-defect ratio for small hernias in rTAPP patients [19, 28]. The downside of rTAPP is that it is difficult to perform in patients with low body fat, but it is useful because future hernias can be treated by a retro muscular approach since the retro rectus plane would be intact [32].

A rare trans-abdominal approach, TA-SM, was ranked last by SUCRA for hospital length of stay reduction. TA-SM is similar to rTARM, with the exception that it may be done in a sublay fashion, which includes both the pre-peritoneal and the retro muscular planes for mesh placement. Based on SUCRA, rTARM was ranked last for seroma formation, which explains its similarly low rank for reoperation rate reduction [36]. The less favorable estimates observed for TA-SM and TARM may reflect procedural complexity, patient selection, and sparse data rather than true comparative inferiority. Mechanistic explanations remain speculative and should not be overinterpreted.

### Limitations of the study

This review has several limitations. First, most included evidence was observational and therefore vulnerable to confounding by indication and selection bias. Second, important clinical heterogeneity existed across studies in hernia type, defect width, reconstructive complexity, and case selection. Third, several techniques were grouped into pooled anatomic nodes, which may have introduced classification uncertainty. In addition, certain approaches — most notably rTAR — are preferentially selected for larger and more complex defects, so their comparative estimates are vulnerable to confounding by indication; although an exploratory analysis excluding rTAR did not materially alter the principal comparisons, residual case-mix imbalance across nodes cannot be fully excluded. Fourth, follow-up duration, follow-up completeness, and recurrence ascertainment were inconsistently reported, limiting confidence in short-term recurrence comparisons. Fifth, defect closure status — anterior fascial closure (IPOM-plus), partial closure, or bridged repair — was not uniformly reported across studies. Our systematic re-extraction revealed that rIPOM arms showed the widest variation in closure rates (60–88% in observational cohorts; 100% in protocol-mandated trials), whereas all preperitoneal and retromuscular approaches achieved 92–100% closure. Because defect closure independently influences seroma formation, short-term recurrence, and overall complication rates, this differential practice may have partially attenuated the apparent inferiority of rIPOM relative to preperitoneal approaches in the current network. Standardised reporting of fascial closure status — with distinction between IPOM-plus, partial closure, and bridged repair — is strongly recommended as a minimum dataset element for future comparative studies in robotic ventral hernia repair.

### Recommendations for future research

Given that only 200 of 9,348 included patients were randomized, the most informative next step would likely be a definitive multicenter RCT comparing rIPOM with eTEP in small-to-medium midline ventral or incisional hernias. [Inference] Using the current network recurrence signal as an illustrative assumption, such a trial would require approximately 220 patients per group, or about 240 per group allowing for attrition, with standardized recurrence assessment and follow-up extending beyond 1 year.

## Conclusion

In this network meta-analysis, preperitoneal mesh placement was associated with more favorable estimates for several short-term postoperative outcomes than intraperitoneal onlay mesh (rIPOM); this preperitoneal-versus-intraperitoneal contrast represents the most consistent and interpretable signal in the data. Technique-level differences, including those involving rTEP-RM and rTAPP, were directionally supportive but were derived from sparser, exploratory comparisons. Because most comparisons were derived from observational studies and supported by low or very low certainty evidence, these findings should be interpreted as exploratory and hypothesis-generating rather than as definitive evidence of superiority. The principal contribution of this work is to map the current comparative evidence across robotic techniques and to define the most informative next step: a definitive multicenter randomized trial comparing rIPOM with eTEP in small-to-medium midline ventral and incisional hernias, employing standardized recurrence ascertainment and follow-up extending beyond one year.

## Electronic Supplementary Material

Below is the link to the electronic supplementary material.

Supplementary file1 (DOCX 6.96 MB)

Supplementary file2 Risk of bias of RCTs (PDF 154 KB)

Supplementary file3 Risk of bias of observational studies (PDF 232 KB)

Supplementary file4 Forest plot meta-analysis for reoperation rate, console time and total operative time (PDF 185 KB)

Supplementary file5 Network plots from the meta-analysis for a specific outcome (e.g., reoperation rate, total operative time, console time,and intraoperative complication rate (PDF 150 KB)

Supplementary file6 Forest plot meta-analysis for hematoma rate and intraoperative complications rate (PDF 167 KB)

Supplementary file7 Network plots from the meta-analysis for a specific outcome (e.g., hematoma rate) (PDF 58 KB)

## Data Availability

All data generated or analyzed during this study are included in the published article.

## Abbreviations

BMI: Body mass index
CENTRAL: Cochrane central register of controlled trials
CI: Confidence interval
eTAPP: Enhanced-view transabdominal preperitoneal
eTEP: Enhanced-view totally extraperitoneal
FFC: Freedom from complication
I^2^: I-squared (heterogeneity statistic)
IH: Incisional hernia
IPOM: Intraperitoneal onlay mesh
IQR: Interquartile range
LOS: Length of stay/length of hospital stay
MD: Mean difference
MeSH: Medical subject headings
MIS: Minimally invasive surgery
NMA: Network meta-analysis
PICO: Population, intervention, comparator, outcome
PP: Preperitoneal (group)
PRISMA: Preferred reporting items for systematic reviews and meta-analyses
PROSPERO: International prospective register of systematic reviews
PRS: Posterior rectus sheath
RCT: Randomized controlled trial
rIPOM: Robotic intraperitoneal onlay mesh
RM: Retromuscular (group)
RoB 2: Cochrane risk of bias 2
ROBINS-I: Risk of bias in non-randomized studies of interventions
RR: Risk ratio
rRM: Robotic retromuscular
rRS: Robotic retrorectus sublay
rTA-RM: Robotic transabdominal retromuscular
rTAPP: Robotic transabdominal preperitoneal
rTAR: Robotic transversus abdominis release
rTEP-RM: Robotic totally extraperitoneal retromuscular
RVHR: Robotic ventral hernia repair
SD: Standard deviation
SE: Standard error
SMD: Standardized mean difference
SRMA: Systematic review and meta-analysis
SSI: Surgical site infection
SUCRA: Surface under the cumulative ranking curve
TA-SM: Transabdominal sublay mesh
TAR: Transversus abdominis release
TARM: Transabdominal retromuscular
US: United States
VH: Ventral hernia
VHR: Ventral hernia repair
τ^2^: Tau-squared (between-study variance)
