# Supplementary material for: Network meta-analysis of robotic ventral and incisional hernia repair techniques: intraperitoneal, preperitoneal, retromuscular, and sublay approaches
Source: Hernia. 2026 Oct 1;30(1):358. doi: 10.1007/s10029-026-03828-y (PMC13630948; doi:10.1007/s10029-026-03828-y)

Risk of Bias 2 Assessment of Randomized Controlled Trials

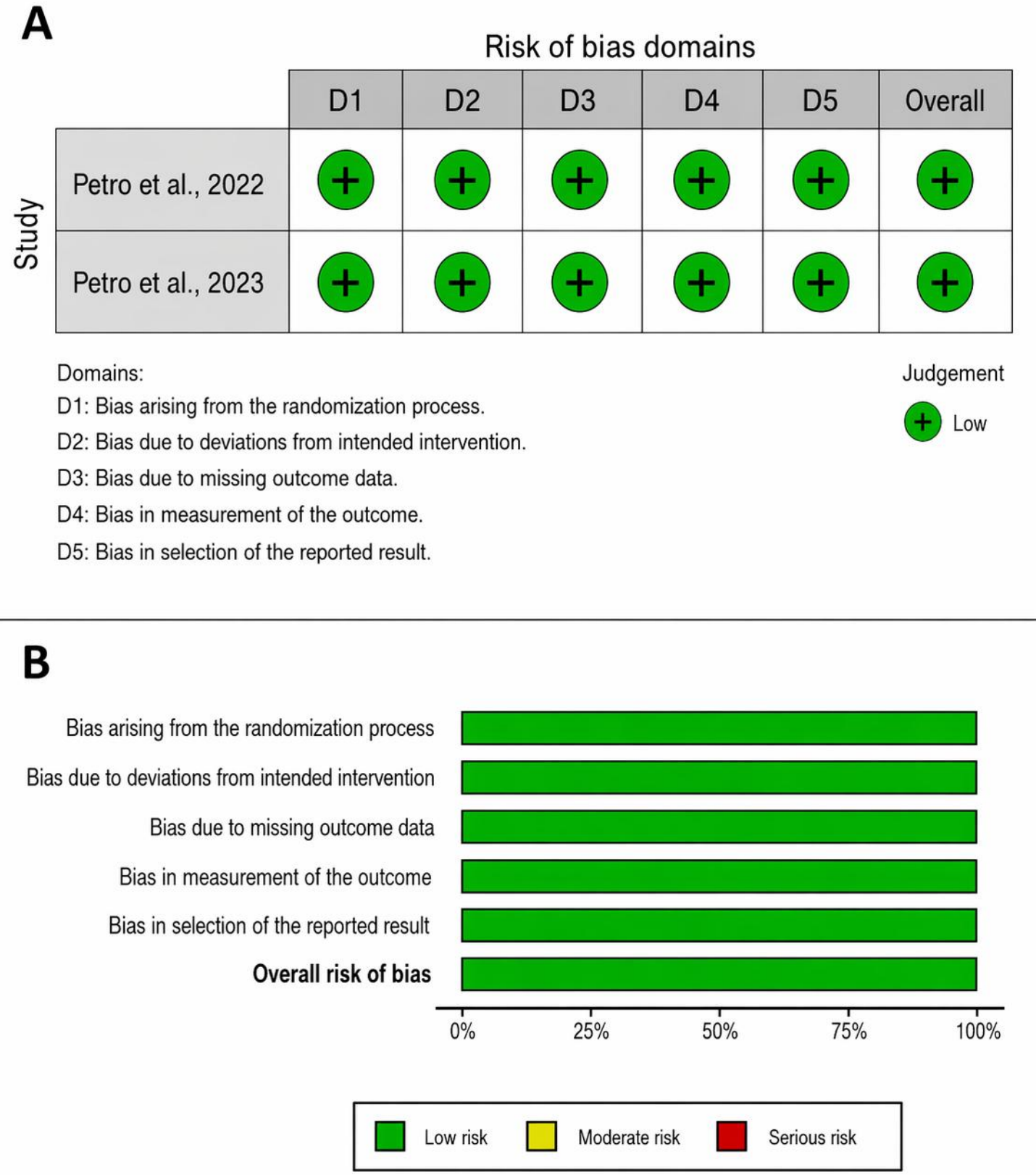

Supplement: Supplementary file 1 [file 10029_2026_3828_MOESM2_ESM.pdf]
