# Supplementary material for: Network meta-analysis of robotic ventral and incisional hernia repair techniques: intraperitoneal, preperitoneal, retromuscular, and sublay approaches
Source: Hernia. 2026 Oct 1;30(1):358. doi: 10.1007/s10029-026-03828-y (PMC13630948; doi:10.1007/s10029-026-03828-y)

Comparison: other vs 'rIPOM' (Common Effects Model)

Treatment	RR	95%-CI	pval	SUCRA
PP	0.45	[0.19; 1.03]	0.060	0.80
rTEP-RM	0.43	[0.07; 2.82]	0.378	0.77
rTAPP	0.65	[0.01; 28.08]	0.821	0.62
rIPOM	1.00		.	0.56
rRM	1.22	[0.06; 25.63]	0.897	0.49
eTEP	1.73	[0.24; 12.64]	0.587	0.41
rTA-RM	2.14	[0.06; 75.34]	0.675	0.40
RM	2.60	[1.50; 4.49]	< 0.001	0.28
TARM	8.62	[0.20; 364.59]	0.259	0.17

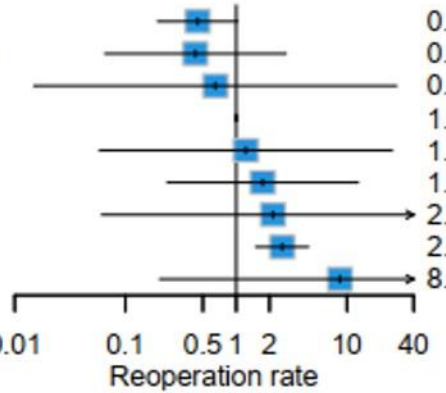

Comparison: other vs 'rIPOM' (Random Effects Model)

Treatment	MD	95%-CI	pval	SUCRA
rIPOM	0.00		.	0.92
rTAPP	2.13	[-8.27; 12.54]	0.688	0.87
rTEP-RM	17.33	[-7.15; 41.81]	0.165	0.63
rRS	25.94	[9.45; 42.43]	0.002	0.51
rTA-RM	53.17	[12.90; 93.44]	0.010	0.29
rRM	63.23	[13.06; 113.40]	0.014	0.26
rTAR	141.11	[118.65; 163.57]	< 0.001	0.00

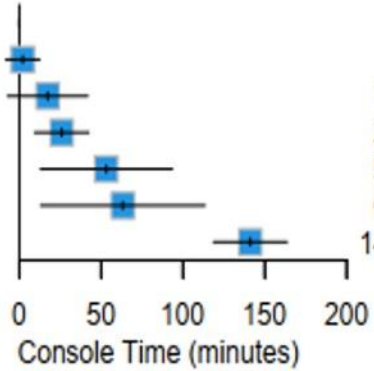

Comparison: other vs 'rIPOM' (Random Effects Model)

Treatment	MD	95%-CI	pval	SUCRA
rTAPP	-1.12	[-8.66; 6.42]	0.771	0.95
rIPOM	0.00		.	0.94
rTEP-RM	16.00	[-3.69; 35.69]	0.111	0.77
rRS	26.32	[12.68; 39.97]	< 0.001	0.68
rRM	35.62	[20.78; 50.46]	< 0.001	0.56
TA-SM	50.46	[16.70; 84.22]	0.003	0.38
rTA-RM	50.50	[14.05; 86.95]	0.007	0.37
TARM	52.79	[27.63; 77.94]	< 0.001	0.34
eTEP	52.96	[38.35; 67.56]	< 0.001	0.33
eTAPP	72.96	[33.19; 112.72]	< 0.001	0.18
rTAR	146.15	[124.61; 167.69]	< 0.001	0.00

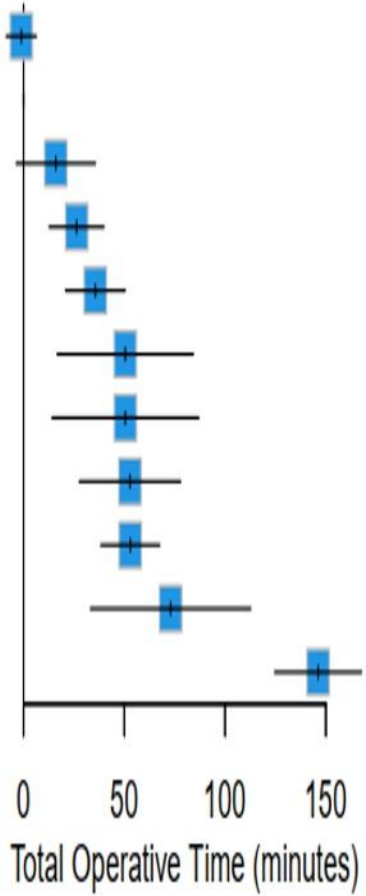

Supplement: Supplementary file 3 [file 10029_2026_3828_MOESM4_ESM.pdf]
