# Supplementary material for: Network meta-analysis of robotic ventral and incisional hernia repair techniques: intraperitoneal, preperitoneal, retromuscular, and sublay approaches
Source: Hernia. 2026 Oct 1;30(1):358. doi: 10.1007/s10029-026-03828-y (PMC13630948; doi:10.1007/s10029-026-03828-y)
Supplement: Supplementary file 4 [file 10029_2026_3828_MOESM5_ESM.pdf]

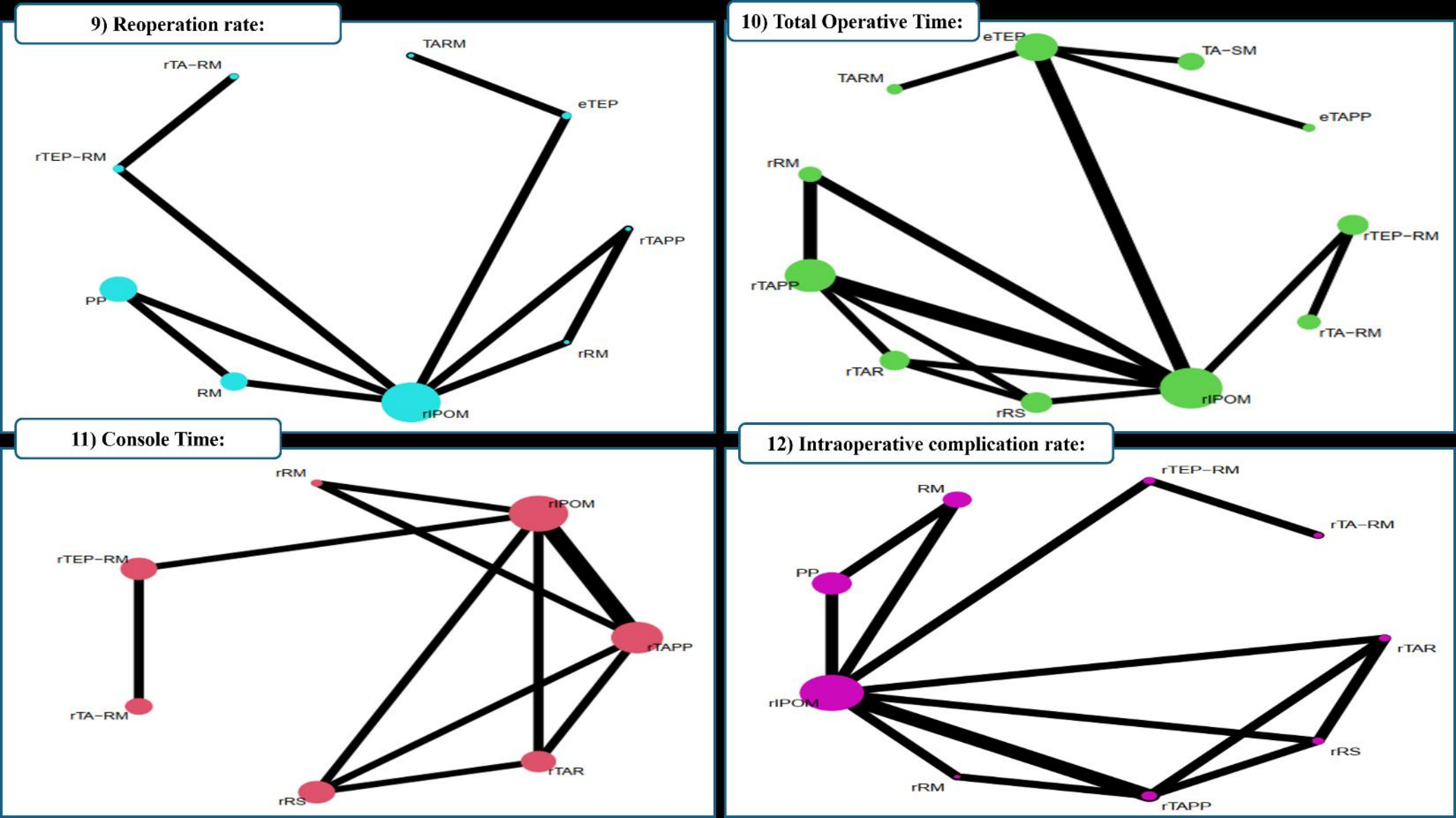
9) Reoperation rate:
TARM
rTA−RM
eTEP
rTEP−RM
rTAPP
PP
rRM
RM
rIPOM
10) Total Operative Time:
eTEP
TA−SM
TARM
eTAPP
rRM
rTEP−RM
rTAPP
rTA−RM
rTAR
rIPOM
rRS
11) Console Time:
rRM
rIPOM
rTEP−RM
rTAPP
rTA−RM
rTAR
rRS
12) Intraoperative complication rate:
rTEP−RM
RM
rTA−RM
PP
rTAR
rIPOM
rRS
rRM
rTAPP
