# Supplementary material for: Network meta-analysis of robotic ventral and incisional hernia repair techniques: intraperitoneal, preperitoneal, retromuscular, and sublay approaches
Source: Hernia. 2026 Oct 1;30(1):358. doi: 10.1007/s10029-026-03828-y (PMC13630948; doi:10.1007/s10029-026-03828-y)
Supplement: Supplementary file 5 [file 10029_2026_3828_MOESM6_ESM.pdf]

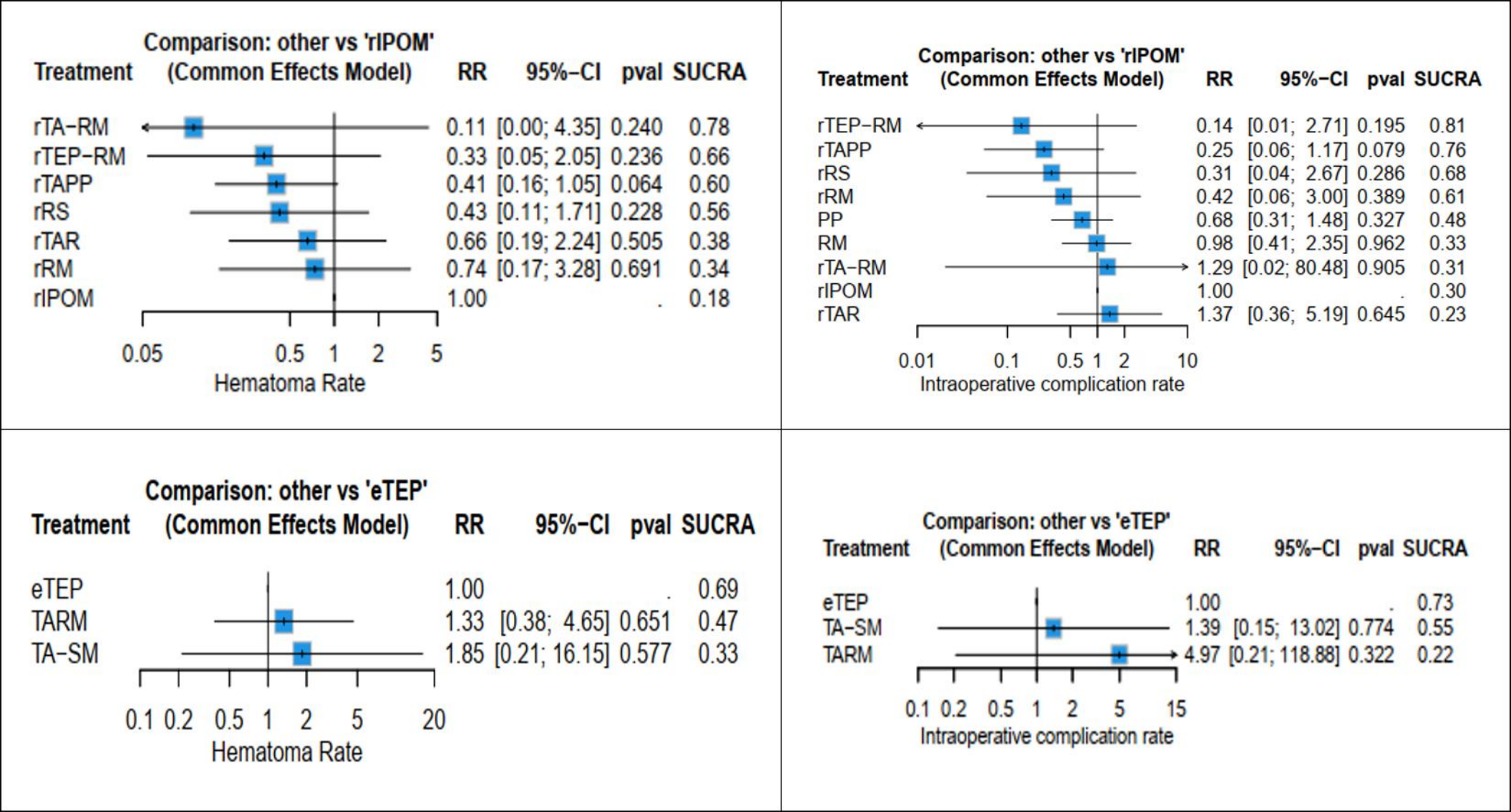
Comparison: other vs 'rIPOM'
Treatment (Common Effects Model) RR 95%-CI pval SUCRA
rTA-RM 0.11 [0.00; 4.35] 0.240 0.78
rTEP-RM 0.33 [0.05; 2.05] 0.236 0.66
rTAPP 0.41 [0.16; 1.05] 0.064 0.60
rRS 0.43 [0.11; 1.71] 0.228 0.56
rTAR 0.66 [0.19; 2.24] 0.505 0.38
rRM 0.74 [0.17; 3.28] 0.691 0.34
rIPOM 1.00 . 0.18
0.05 0.5 1 2 5
Hematoma Rate
Comparison: other vs 'rIPOM'
Treatment (Common Effects Model) RR 95%-CI pval SUCRA
rTEP-RM 0.14 [0.01; 2.71] 0.195 0.81
rTAPP 0.25 [0.06; 1.17] 0.079 0.76
rRS 0.31 [0.04; 2.67] 0.286 0.68
rRM 0.42 [0.06; 3.00] 0.389 0.61
PP 0.68 [0.31; 1.48] 0.327 0.48
RM 0.98 [0.41; 2.35] 0.962 0.33
rTA-RM 1.29 [0.02; 80.48] 0.905 0.31
rIPOM 1.00 . 0.30
rTAR 1.37 [0.36; 5.19] 0.645 0.23
0.01 0.1 0.5 1 2 10
Intraoperative complication rate
Comparison: other vs 'eTEP'
Treatment (Common Effects Model) RR 95%-CI pval SUCRA
eTEP 1.00 . 0.69
TARM 1.33 [0.38; 4.65] 0.651 0.47
TA-SM 1.85 [0.21; 16.15] 0.577 0.33
0.1 0.2 0.5 1 2 5 20
Hematoma Rate
Comparison: other vs 'eTEP'
Treatment (Common Effects Model) RR 95%-CI pval SUCRA
eTEP 1.00 . 0.73
TA-SM 1.39 [0.15; 13.02] 0.774 0.55
TARM 4.97 [0.21; 118.88] 0.322 0.22
0.1 0.2 0.5 1 2 5 15
Intraoperative complication rate
