# Supplementary material for: Network meta-analysis of robotic ventral and incisional hernia repair techniques: intraperitoneal, preperitoneal, retromuscular, and sublay approaches
Source: Hernia. 2026 Oct 1;30(1):358. doi: 10.1007/s10029-026-03828-y (PMC13630948; doi:10.1007/s10029-026-03828-y)
Supplement: Supplementary file 7 [file 10029_2026_3828_MOESM1_ESM.docx]

**Supplementary Tables & Evidence Synthesis**

**Robotic Ventral Hernia Repair — Network Meta-Analysis**

**INDEX OF TABLES**

| **No.** | **Table Title** | **Location** |
| --- | --- | --- |
| **1** | Supplementary Table 1. **Baseline and Hernia Characteristics of Included Studies** | Section 1 |
| **2** | Supplementary Table 2. Full GRADE Evidence Profile for Network Meta-Analysis  — Detailed certainty assessment for all outcomes and comparisons (NMA-GRADE method) | Section 2 |
| **3** | Supplementary Table 3. Follow-up and Recurrence Assessment Summary  — Follow-up duration, completeness, and recurrence ascertainment methods by study | Section 3 (a) |
| **4** | Supplementary Table 4. Study-to-Node Mapping Used for Network Construction  — Original arm labels mapped to final network nodes with rationale | Section 3 (b) |
| **5** | Supplementary 1. PRISMA 2020 Checklist | Section 4 |
| **6** | Supplementary table 5. Database Search Strategies | Section 4 |
| **7** | Supplementary 3. Rankogram | Section 4 |
| **8** | Supplementary 4. League Tables — All Outcomes (1–14)  Hernia recurrence, overall complications, SSI, pain, LOS, seroma, readmission, reoperation,  operative time, console time, hematoma, intraoperative complications | Section 4 |
| **9** | . DEFECT CLOSURE STATUS | Section 5 |

**Section 1**

| **Table 1. Baseline and Hernia Characteristics of Included Studies** | | | | | | | | | | | | | | | | | | | | |
| --- | --- | --- | --- | --- | --- | --- | --- | --- | --- | --- | --- | --- | --- | --- | --- | --- | --- | --- | --- | --- |
| **Study Information** | | | | | **Patient Characteristics** | | | | **Hernia Morphology** | | | | | **Hernia Location** | | **Hernia Etiology** | | | | |
| **Study ID** | **Arm** | **Design** | **Year** | **Setting** | **N** | **Age (Mean±SD)** | **Female n (%)** | **BMI (Mean±SD)** | **Defect Width (cm)** | **Defect Length (cm)** | **Mesh Size (cm²)** | **Overlap (cm)** | **Defect Area (cm²)** | **Midline n (%)** | **Lateral n (%)** | **Umbilical n (%)** | **Incisional n (%)** | **Epigastric n (%)** | **Ventral n (%)** | **Other / NR** |
| **Kudsi et al 2020 (TA-RM vs TEP/RM)** | TA-RM | Retro. cohort | 2020 | Elective | 82 | 57.8±13.1 | 60 (54.1%) | 32.6±6.5 | — | — | 393.3±301.9 | 6.83±2.26 | 34.5±46.2 | — | — | — | 54 (65.9%) | — | 28 (34.1%) | — |
|  | TEP/RM |  |  |  | 82 | 56.6±14.7 | 46 (44.7%) | 31.3±6.7 | — | — | 375±282.9 | 6.5±1.5 | 35.6±47.5 | — | — | — | 50 (61%) | — | 32 (39%) | — |
| **Kudsi et al 2021** | IPOM | Retro. cohort | 2021 | Elective | 90 | 47.9±14.5 | 48 (53.3%) | 31.3±6.3 | — | — | 96.5±37.2 | 4±1.1 | 4.1±2.3 | — | — | 83 (92.2%) | — | 9 (10%) | — | — |
|  | TAPP |  |  |  | 108 | 50.2±14.2 | 30 (27.8%) | 30.5±5.7 | — | — | 117.9±85.2 | 4.3±1.1 | 4.4±2.9 | — | — | 104 (96.3%) | — | 12 (11.1%) | — | — |
|  | RM |  |  |  | 71 | 52±13.7 | 26 (36.6%) | 30±6.4 | — | — | 250±56.8 | 5.7±0.4 | 13.5±8.5 | — | — | 70 (98.6%) | — | 14 (19.7%) | — | — |
| **Kudsi et al 2022** | IPOM | Retro. cohort | 2022 | Elective | 197 | 54.3±14.4 | 109 (55.3%) | 32.6±6.7 | 2.5±1.1 | — | 108.9±64.5 | 4.2±1.1 | 6.9±7.1 | 191 (97%) | 6 (3%) | — | — | — | 108 (54.8%) | — |
|  | TAPP |  |  |  | 156 | 52.1±14.3 | 53 (34%) | 31.9±6.2 | 2.3±0.75 | — | 118.9±87.1 | 4.3±1.1 | 4.5±3.2 | 141 (90.4%) | 13 (8.3%) | — | — | — | 117 (75%) | — |
|  | RS |  |  |  | 153 | 54.6±14.2 | 52 (34%) | 31.1±6.5 | 3.7±0.75 | — | 250±56.1 | 5.6±0.15 | 9.8±7.5 | 153 (100%) | 0 (0%) | — | — | — | 108 (70.6%) | — |
|  | TAR |  |  |  | 138 | 60.2±13.2 | 82 (59.4%) | 33.1±7 | 7.3±6 | — | 600±449.5 | 6.7±3 | 69.5±91.5 | 102 (73.9%) | 19 (13.8%) | — | — | — | 15 (10.9%) | — |
| **Kudsi et al 2020 (lateral incisional)** | IPOM | Retro. cohort | 2020 | Elective | 5 | 58.2±6.7 | 3 (60%) | 34.9±2.2 | — | — | 207.3±284.4 | 5.2±1.5 | 25.3±49.8 | — | — | — | — | — | — | — |
|  | TAPP |  |  |  | 8 | 57±13.7 | 5 (62.5%) | 28.9±5 | — | — | 189.5±105.9 | 3.9±2.1 | 11.7±16.8 | — | — | — | — | — | — | — |
|  | RM |  |  |  | 13 | 62.1±15.8 | 10 (76.9%) | 30.5±6 | — | — | 325±186.9 | 5.8±0.8 | 15.7±5.2 | — | — | — | — | — | — | — |
| **Kudsi et al 2020 (IPOM vs TEP-RM)** | IPOM | Retro. cohort | 2020 | Elective | 68 | 57.8±12.7 | 34 (50%) | 31.2±5.7 | — | — | 134.2±48.3 | 4.2±1.1 | 13.6±15.5 | 64 (94.1%) | 4 (5.9%) | — | 40 (58.8%) | — | 28 (41.2%) | — |
|  | TEP-RM |  |  |  | 68 | 56.5±15.9 | 32 (47.1%) | 31.6±7 | — | — | 300±113.6 | 5.7±0.4 | 16.97±8.9 | 59 (86.8%) | 9 (13.2%) | — | 28 (41.2%) | — | 40 (58.8%) | — |
| **Al-Mansour et al 2022** | IPOM | Retro. cohort | 2022 | Elective | 4486 | 56.8±15.28 | 2235 (49.8%) | 32.33±6.67 | 3.0±1.5 | 3.7±3 | 131.4±64.1 | — | — | 4208 (93.9%) | 413 (9.2%) | 1284 (28.6%) | 2832 (63.2%) | 393 (8.8%) | — | — |
|  | PP |  |  |  | 1829 | 54±16.32 | 781 (42.7%) | 31.70±6.23 | 2.3±0.7 | 3.0±1.5 | 114.1±53.6 | — | — | 1581 (86.5%) | 283 (15.5%) | 873 (47.7%) | 680 (37.2%) | 226 (12.4%) | — | — |
|  | RM |  |  |  | 948 | 56.33±14.85 | 496 (52.3%) | 31.90±6.24 | 4.3±1.5 | 7±5.2 | 311.6±169.1 | — | — | 902 (95.2%) | 100 (10.6%) | 187 (19.7%) | 690 (72.8%) | 103 (10.9%) | — | — |
| **Gokcal et al 2019** | IPOM | Retro. cohort | 2019 | Elective | 104 | 50.9±14.5 | 44 (42.3%) | 31.22±5.60 | 2.3±0.75 | 2.3±0.75 | 96.6±37.2 | 4.2±1.1 | 4.5±2.95 | 99 (95.2%) | 5 (4.8%) | — | 29 (27.9%) | — | 75 (72.1%) | — |
|  | TAPP |  |  |  | 104 | 51.2±14.1 | 41 (39.4%) | 31.60±5.22 | 2.3±0.75 | 2.5±0.75 | 118.9±87.5 | 4.3±1.1 | 4.6±2.95 | 99 (94.2%) | 6 (5.8%) | — | 27 (26%) | — | 77 (74%) | — |
| **Kennedy et al 2017** | IPOM | Retro. cohort | 2017 | Elective | 27 | 45.6±13.1 | 22 (81.4%) | 31.8±9.6 | — | — | — | — | 3.40±1.51 | — | — | — | — | — | — | — |
|  | TAPP |  |  |  | 36 | 44.3±13.3 | 24 (66.7%) | 31.0±6.4 | — | — | — | — | 3.98±2.61 | — | — | — | — | — | — | — |
| **Pacheco et al 2024** | TARM | Retro. cohort | 2024 | NR | 36 | 57.47±12.0 | 18 (50%) | 33.24±6.5 | 5.10±1.4 | — | 329.06±70.3 | — | — | — | — | — | 20 (55.5%) | — | 16 (44.4%) | — |
|  | ETEP |  |  |  | 60 | 57.35±11.2 | 32 (53.3%) | 30.47±4.7 | 4.9±2.6 | — | 724.56±193.7 | — | — | — | — | — | 32 (53.3%) | — | 28 (46.6%) | — |
| **Petro et al 2023 (REVEAL 1-year)** | ETEP | RCT | 2023 | Elective | 51 | 51.90±16.25 | 22 (45%) | 33.76±5.15 | 4±1.5 | 6±5.3 | 360.3±121.3 | — | — | — | — | — | — | — | — | — |
|  | IPOM |  |  |  | 49 | 57.23±15.50 | 25 (49%) | 30.86±7.36 | 3.50±2.3 | 4.8±4.8 | 200±57.3 | — | — | — | — | — | — | — | — | — |
| **Pini et al 2024** | ETEP | Retro. cohort | 2024 | NR | 32 | 57.8±11.9 | 5 (15.6%) | 27.1±5.5 | — | — | 214±42 | — | 2.5±0.9 | — | — | 27 (84.4%) | 7 (21.9%) | 2 (6.2%) | — | — |
|  | ETAPP |  |  |  | 21 | 60.9±16.6 | 3 (14.2%) | 29.5±6.3 | — | — | 211±245 | — | 2.5±0.8 | — | — | 16 (76.2%) | 2 (9.5%) | 2 (9.5%) | — | — |
| **Saleh et al 2025** | IPOM | Retro. cohort | 2025 | NR | 44 | 57.4±14.6 | 22 (50.0%) | 36.0±9.4 | — | — | — | 34.7±51.4 | — | — | — | — | — | — | — | — |
|  | ETEP |  |  |  | 44 | 56.8±13.7 | 18 (40.9%) | 34.2±7.7 | — | — | — | 40.7±41.4 | — | — | — | — | — | — | — | — |
| **Zaman et al 2023** | ETEP | Retro. cohort | 2023 | Elective | 50 | 53.5±13.6 | 31 (62%) | 33.1±7.8 | — | — | 432.8±246.4 | 109.1±148.5 | — | — | — | — | 45 (90%) | — | 5 (10%) | — |
|  | TA-SM |  |  |  | 108 | 51.0±12.7 | 67 (62%) | 33.7±7.7 | — | — | 137.9±146.8 | 31.8±59.3 | — | — | — | — | 98 (90.7%) | — | 10 (9.26%) | — |
| **Petro et al 2022 (REVEAL 30-day)** | IPOM | RCT | 2022 | Elective | 49 | 57.23±15.50 | 22 (45%) | 30.86±7.36 | 3.50±2.3 | 4.8±4.8 | 200±57.3 | — | — | — | — | — | — | — | — | — |
|  | ETEP |  |  |  | 51 | 52.90±16.25 | 25 (49%) | 33.76±5.15 | 4±1.5 | 6±5.3 | 360.3±121.3 | — | — | — | — | — | — | — | — | — |

Abbreviations: Retro. = retrospective; RCT = randomised controlled trial; NR = not reported; NA = not available; TA-RM = transabdominal retromuscular; TEP/RM = totally extraperitoneal retromuscular; IPOM = intraperitoneal onlay mesh; TAPP = transabdominal preperitoneal; RS = robotic surgery; TAR = transversus abdominis release; RM = retromuscular; PP = prepertional ; TARM = transabdominal retromuscular; ETEP = enhanced view totally extraperitoneal; ETAPP = enhanced view transabdominal preperitoneal; TA-SM = transabdominal sublay mesh; TEP-RM = totally extraperitoneal retromuscular. Values presented as mean ± SD unless otherwise stated. — = not reported.

**Section 2**

| **Table 2. Full GRADE Evidence Profile — Network Meta-Analysis of Robotic Ventral Hernia Repair Techniques vs rIPOM** | | | | | | | | | | |
| --- | --- | --- | --- | --- | --- | --- | --- | --- | --- | --- |
| **Outcome & Comparison** | | | **Risk of Bias** | | **GRADE Domains** | | | | **Summary** | |
| **Outcome** | **Comparison**  **(vs rIPOM)** | **Relative Effect**  **(95% CI)** | **Within-study Bias** | **Reporting Bias** | **Indirectness** | **Imprecision** | **Heterogeneity** | **Incoherence** | **Overall Certainty**  **(GRADE)** | **Comments** |
| **HERNIA RECURRENCE, 1 YEAR** | | | | | | | | | | |
|  | PP vs rIPOM | RR 0.50 (0.43–0.58) | Major concerns | Some concerns | No concerns | Some concerns | No concerns | No concerns | **⨁⨁◯◯**  **LOW** | Downgraded 2 for RoB (observational cohorts); 1 for imprecision (limited events). |
|  | rTAPP vs rIPOM | RR 0.55 (0.36–0.86) | Major concerns | Some concerns | No concerns | Some concerns | No concerns | No concerns | **⨁⨁◯◯**  **LOW** | Downgraded 2 for RoB; 1 for imprecision (CI includes small and moderate benefit). |
|  | rRS vs rIPOM | RR 0.50 (0.30–0.85) | Major concerns | Some concerns | No concerns | Some concerns | No concerns | No concerns | **⨁⨁◯◯**  **LOW** | Downgraded 2 for RoB; 1 for imprecision (relatively few events). |
|  | rTAR vs rIPOM | RR 2.71 (2.03–3.62) | Major concerns | Some concerns | No concerns | No concerns | No concerns | No concerns | **⨁⨁◯◯**  **LOW** | Downgraded 2 for RoB; confounding by indication likely. |
|  | eTEP vs rIPOM | RR 0.77 (0.43–1.38) | Major concerns | Some concerns | No concerns | Major concerns | No concerns | No concerns | **⨁◯◯◯ VERY LOW** | Downgraded 2 for RoB; 2 for imprecision (wide CI, crosses null). |
|  | RM vs rIPOM | RR 0.94 (0.82–1.07) | Major concerns | Some concerns | No concerns | Major concerns | No concerns | No concerns | **⨁◯◯◯ VERY LOW** | Downgraded 2 for RoB; 2 for imprecision (sparse data, CI includes benefit and harm). |
|  | rRM vs rIPOM | RR 1.54 (0.70–3.36) | Major concerns | Some concerns | No concerns | Major concerns | No concerns | No concerns | **⨁◯◯◯ VERY LOW** | Downgraded 2 for RoB; 2 for imprecision (very wide CI). |
|  | rTA-RM vs rIPOM | RR 0.91 (0.57–1.44) | Major concerns | Some concerns | No concerns | Major concerns | No concerns | No concerns | **⨁◯◯◯ VERY LOW** | Downgraded 2 for RoB; 2 for imprecision (wide CI, crosses null). |
|  | rTEP-RM vs rIPOM | RR 0.63 (0.35–1.12) | Major concerns | Some concerns | No concerns | Major concerns | No concerns | No concerns | **⨁◯◯◯ VERY LOW** | Downgraded 2 for RoB; 2 for imprecision (CI includes both benefit and no effect). |
|  | TA-SM vs rIPOM | RR 0.55 (0.28–1.08) | Major concerns | Some concerns | No concerns | Major concerns | No concerns | No concerns | **⨁◯◯◯ VERY LOW** | Downgraded 2 for RoB; 2 for imprecision (sparse data, wide CI). |
|  | TARM vs rIPOM | RR 0.72 (0.40–1.31) | Major concerns | Some concerns | No concerns | Major concerns | No concerns | No concerns | **⨁◯◯◯ VERY LOW** | Downgraded 2 for RoB; 2 for imprecision (wide CI crosses null). |
| **OVERALL COMPLICATIONS, 30 DAYS** | | | | | | | | | | |
|  | rTEP-RM vs rIPOM | RR 0.23 (0.07–0.74) | Major concerns | Some concerns | No concerns | Some concerns | Some concerns | No concerns | **⨁⨁◯◯**  **LOW** | Downgraded 2 for RoB; 1 for imprecision (few studies and events). |
|  | rTAPP vs rIPOM | RR 0.55 (0.30–0.99) | Major concerns | Some concerns | No concerns | Some concerns | No concerns | No concerns | **⨁⨁◯◯**  **LOW** | Downgraded 2 for RoB; 1 for imprecision (CI includes little or no benefit). |
|  | PP vs rIPOM | RR 0.67 (0.45–0.99) | Major concerns | Some concerns | No concerns | No concerns | No concerns | No concerns | **⨁⨁◯◯**  **LOW** | Downgraded 2 for RoB; imprecision not serious. |
|  | RM vs rIPOM | RR 0.65 (0.50–0.84) | Major concerns | Some concerns | No concerns | No concerns | No concerns | No concerns | **⨁⨁◯◯**  **LOW** | Downgraded 2 for RoB; imprecision not serious. |
|  | rRM vs rIPOM | RR 1.06 (0.43–2.61) | Major concerns | Some concerns | No concerns | Major concerns | No concerns | No concerns | **⨁◯◯◯ VERY LOW** | Downgraded 2 for RoB; 2 for imprecision (very wide CI). |
|  | rRS vs rIPOM | RR 1.08 (0.61–1.90) | Major concerns | Some concerns | No concerns | Major concerns | No concerns | No concerns | **⨁◯◯◯ VERY LOW** | Downgraded 2 for RoB; 2 for imprecision (wide CI). |
|  | rTAR vs rIPOM | RR 2.97 (1.82–4.85) | Major concerns | Some concerns | No concerns | Some concerns | No concerns | No concerns | **⨁⨁◯◯**  **LOW** | Downgraded 2 for RoB; effect large; confounding by indication. |
|  | eTEP vs rIPOM | RR 1.62 (0.69–3.79) | Major concerns | Some concerns | No concerns | Major concerns | No concerns | No concerns | **⨁◯◯◯ VERY LOW** | Downgraded 2 for RoB; 2 for imprecision (wide CI). |
|  | eTAPP vs rIPOM | RR 1.14 (0.26–5.04) | Major concerns | Some concerns | No concerns | Major concerns | No concerns | No concerns | **⨁◯◯◯ VERY LOW** | Downgraded 2 for RoB; 2 for imprecision. |
|  | TA-SM vs rIPOM | RR 1.33 (0.60–2.96) | Major concerns | Some concerns | No concerns | Major concerns | No concerns | No concerns | **⨁◯◯◯ VERY LOW** | Downgraded 2 for RoB; 2 for imprecision. |
| **OVERALL SURGICAL-SITE INFECTION (SSI), 30 DAYS** | | | | | | | | | | |
|  | rRS vs rIPOM | RR 0.11 (0.02–0.87) | Major concerns | Some concerns | No concerns | Some concerns | No concerns | No concerns | **⨁⨁◯◯**  **LOW** | Downgraded 2 for RoB; 1 for imprecision (very few events, wide CI). |
|  | rTAPP vs rIPOM | RR 0.32 (0.12–0.86) | Major concerns | Some concerns | No concerns | Some concerns | No concerns | No concerns | **⨁⨁◯◯**  **LOW** | Downgraded 2 for RoB; 1 for imprecision. |
|  | PP vs rIPOM | RR 0.44 (0.24–0.79) | Major concerns | Some concerns | No concerns | No concerns | No concerns | No concerns | **⨁⨁◯◯**  **LOW** | Downgraded 2 for RoB; imprecision not serious. |
|  | rRM vs rIPOM | RR 0.87 (0.08–9.71) | Major concerns | Some concerns | No concerns | Major concerns | No concerns | No concerns | **⨁◯◯◯ VERY LOW** | Downgraded 2 for RoB; 2 for imprecision (very wide CI). |
|  | RM vs rIPOM | RR 1.35 (0.80–2.30) | Major concerns | Some concerns | No concerns | Major concerns | No concerns | No concerns | **⨁◯◯◯ VERY LOW** | Downgraded 2 for RoB; 2 for imprecision. |
|  | rTAR vs rIPOM | RR 2.69 (1.60–4.52) | Major concerns | Some concerns | No concerns | Major concerns | No concerns | No concerns | **⨁◯◯◯ VERY LOW** | Downgraded 2 for RoB; confounding by indication. |
| **REOPERATION, 30 DAYS** | | | | | | | | | | |
|  | PP vs rIPOM | RR 0.45 (0.22–0.91) | Major concerns | Some concerns | No concerns | Major concerns | No concerns | No concerns | **⨁⨁◯◯**  **LOW** | Downgraded 2 for RoB; 1 for imprecision (few events). |
|  | RM vs rIPOM | RR 2.60 (1.23–5.49) | Major concerns | Some concerns | No concerns | Some concerns | No concerns | No concerns | **⨁⨁◯◯**  **LOW** | Downgraded 2 for RoB; 1 for imprecision. |
| **SEROMA FORMATION, 30 DAYS** | | | | | | | | | | |
|  | rTEP-RM vs rIPOM | RR 0.28 (0.10–0.80) | Major concerns | Some concerns | No concerns | Some concerns | No concerns | No concerns | **⨁◯◯◯ VERY LOW** | Downgraded 2 for RoB; 1 for imprecision (few events). |
| **TOTAL OPERATIVE TIME (MINUTES)** | | | | | | | | | | |
|  | rTAPP vs rIPOM | MD 1.12 (−6.42 to 8.66) | Major concerns | Some concerns | No concerns | Major concerns | No concerns | No concerns | **⨁◯◯◯ VERY LOW** | Downgraded 2 for RoB; 2 for imprecision (CI crosses null). |
|  | rTEP-RM vs rIPOM | MD 16.00 (0.41–31.59) | Major concerns | Some concerns | No concerns | Some concerns | No concerns | No concerns | **⨁⨁◯◯**  **LOW** | Downgraded 2 for RoB; 1 for imprecision (CI barely excludes null). |
|  | rRM vs rIPOM | MD 35.05 (24.39–45.71) | Major concerns | Some concerns | No concerns | No concerns | No concerns | No concerns | **⨁⨁◯◯**  **LOW** | Downgraded 2 for RoB; imprecision not serious. |
|  | rRS vs rIPOM | MD 28.28 (20.18–36.39) | Major concerns | Some concerns | No concerns | No concerns | No concerns | No concerns | **⨁⨁◯◯**  **LOW** | Downgraded 2 for RoB; imprecision not serious. |
|  | rTA-RM vs rIPOM | MD 50.50 (18.26–82.74) | Major concerns | Some concerns | No concerns | Some concerns | No concerns | No concerns | **⨁⨁◯◯**  **LOW** | Downgraded 2 for RoB; 1 for imprecision (wide CI). |
|  | rTAR vs rIPOM | MD 148.11 (129.58–166.65) | Major concerns | Some concerns | No concerns | No concerns | No concerns | No concerns | **⨁⨁◯◯**  **LOW** | Downgraded 2 for RoB; imprecision not serious; effect large and consistent. |
|  | eTEP vs rIPOM | MD 53.31 (40.52–66.11) | Major concerns | Some concerns | No concerns | No concerns | No concerns | No concerns | **⨁⨁◯◯**  **LOW** | Downgraded 2 for RoB; imprecision not serious. |
|  | eTAPP vs rIPOM | MD 73.31 (36.07–110.56) | Major concerns | Some concerns | No concerns | Some concerns | No concerns | No concerns | **⨁⨁◯◯**  **LOW** | Downgraded 2 for RoB; 1 for imprecision (wide CI). |
|  | TA-SM vs rIPOM | MD 50.81 (20.06–81.56) | Major concerns | Some concerns | No concerns | Some concerns | No concerns | No concerns | **⨁⨁◯◯**  **LOW** | Downgraded 2 for RoB; 1 for imprecision (wide CI). |
|  | TARM vs rIPOM | MD 53.14 (32.20–74.09) | Major concerns | Some concerns | No concerns | Some concerns | No concerns | No concerns | **⨁⨁◯◯**  **LOW** | Downgraded 2 for RoB; 1 for imprecision (wide CI). |

Abbreviations: RR = risk ratio; MD = mean difference; CI = 95% credible interval; RoB = risk of bias; SSI = surgical site infection; rIPOM = robotic intraperitoneal onlay mesh. "Major concerns" highlighted in red. Certainty rated per NMA-GRADE domains: within-study bias, reporting bias, indirectness, imprecision, heterogeneity, incoherence. All comparisons are vs rIPOM as the reference node.

**Section 3 (a)**

| **Table 3 . Follow-up and Recurrence Assessment Summary** | | | | | |
| --- | --- | --- | --- | --- | --- |
| **Study** | **Follow-up Duration** | **Follow-up Completeness** | **Recurrence Assessment Method** | **Time Point Most Relevant to NMA** |  |
| **Al-Mansour 2022** | Median 21 days (IQR 14–37) | Patients lacking 30-day follow-up were excluded | Short-term chart/registry follow-up; no meaningful 1-year recurrence method for this analysis | 30-day only |  |
| **Gokcal 2019** | Clinical visits at 3 weeks and 3 months | 90.4% seen within 3 weeks; 126/208 (60.6%) at <3 months | Clinical follow-up visits; no recurrence observed during study follow-up | 3 months |  |
| **Kennedy 2017** | 30-day follow-up only | Eligibility required 30-day follow-up; exact completeness not otherwise detailed | Recurrence considered among 30-day complications only | 30 days |  |
| **Petro 2022 (REVEAL 30-day)** | Postoperative day 0, day 1, day 7, and day 30 | NR in a way useful for 1-year recurrence | 30-day RCT outcomes; recurrence not the long-term endpoint in this paper | 30 days |  |
| **Petro 2023 (REVEAL 1-year)** | Median 12 months (IQR 11–13) | 7% lost to follow-up | Hernia Recurrence Inventory screening; clinical exam or CT superseded HRI when available | 1 year |  |
| **Pini 2024** | Mean 11.3 ± 6.4 months (eTEP); 20.5 ± 9.7 months (eTAPP) | NR | Follow-up reported, but explicit recurrence-assessment method not clearly detailed | Closest available follow-up |  |
| **Saleh 2025** | Follow-up duration not clearly reported in the extracted full text | NR | Recurrence reported, but exact ascertainment method and follow-up window not clearly specified | Unclear |  |
| **Zaman 2023** | 30-day and 1-year outcomes reported | >1-year follow-up in 43/50 eTEP (86%) and 106/108 TA-SM (98%) | Symptoms concerning recurrence plus physical examination and/or imaging; CT data and chart review used | 1 year |  |
| **Pacheco 2024** | Long-term subgroup average 27 months | Approximately 40% follow-up after 90 days | Physical examination in long-term followed patients; CT imaging in 50% of that subgroup | >90 days / long-term subgroup |  |
| **Kudsi 2020 (TA-RM vs TEP/RM)** | Average 14.6 months (range 1–47.9) | NR | Follow-up up to 90 days for complications; explicit recurrence method not clearly extracted | Follow-up beyond 1 year exists, but recurrence ascertainment unclear |  |
| **Kudsi 2021** | Routine schedule: 3 weeks, 3 months, 6 months, 1 year, then yearly; all patients assessed within 90 days | All patients assessed by telehealth or clinic within 90 days | No recurrence within 90-day follow-up; long-term recurrence method not clearly presented for this paper | 90 days |  |
| **Kudsi 2022 (7-year experience)** | Mean postoperative follow-up 36.1 months | 611 completed follow-up; 33 lost; 15 deaths reported | Ventral Hernia Recurrence Inventory with phone screening; suspected recurrences confirmed by clinical exam or imaging | Closest to 1 year from longitudinal follow-up / long-term KM data |  |
| **Kudsi 2020 (lateral incisional)** | Mean 33.3 months (range 2.9–76.2) | VHRI completed in 22/26 patients (84.6%) | Phone survey using VHRI; positive screens invited for office exam and imaging | Mid-term follow-up |  |
| **Kudsi 2020 (rIPOM vs rTEP-RM)** | Minimum 90-day follow-up chosen; records and phone data reviewed | NR | Designed primarily for early postoperative complications; no clear long-term recurrence method in extracted text | 90 days |  |

Colour coding: Green = Yes (included in primary recurrence synthesis); Red = No; Amber = Unclear or conditional. NMA = network meta-analysis; HRI = Hernia Recurrence Inventory; VHRI = Ventral Hernia Recurrence Inventory; KM = Kaplan-Meier; NR = not reported.

**Section 3 (b)**

| **Table 4. Study-to-Node Mapping Used for Network Construction** | | | |
| --- | --- | --- | --- |
| **Study** | **Original Arm Label** | **Final Network Node** | **Rationale for Assignment** |
| **Kudsi 2020 (TA vs TEP retromuscular)** | TA-RM | **rTA-RM** | Explicitly reported as transabdominal retromuscular repair |
| Kudsi 2020 (TA vs TEP retromuscular) | TEP/RM | **rTEP-RM** | Explicitly reported as totally extraperitoneal retromuscular repair |
| **Kudsi 2021** | IPOM | **rIPOM** | Standard named intraperitoneal onlay mesh arm |
| Kudsi 2021 | TAPP | **rTAPP** | Standard named robotic transabdominal preperitoneal arm |
| Kudsi 2021 | RM | **rRM*** | Small named retromuscular arm retained as a distinct robotic retromuscular node |
| **Kudsi 2022 (7-year experience)** | IPOM | **rIPOM** | Standard named intraperitoneal arm |
| Kudsi 2022 (7-year experience) | TAPP | **rTAPP** | Standard named robotic TAPP arm |
| Kudsi 2022 (7-year experience) | RS | **rRS*** | Likely corresponds to robotic Rives-Stoppa / retrorectus sublay arm |
| Kudsi 2022 (7-year experience) | TAR | **rTAR** | Standard named robotic TAR arm |
| **Kudsi 2020 (lateral incisional)** | IPOM | **rIPOM** | Standard named intraperitoneal arm |
| Kudsi 2020 (lateral incisional) | TAPP | **PP*** | Functions as broader preperitoneal approach in lateral-incisional setting; pooling into PP avoids implying strict equivalence with named midline rTAPP arms |
| Kudsi 2020 (lateral incisional) | RM | **RM*** | Generic retromuscular arm in a lateral-incisional context; better treated as pooled RM than as a standardized named node |
| **Kudsi 2020 (IPOM vs TEP-RM)** | IPOM | **rIPOM** | Standard named intraperitoneal arm |
| Kudsi 2020 (IPOM vs TEP-RM) | TEP-RM | **rTEP-RM** | Standard named robotic TEP-retromuscular arm |
| **Al-Mansour 2022** | IPOM | **rIPOM** | Standard named intraperitoneal arm |
| Al-Mansour 2022 | RT | **PP*** | [Inference] Appears to represent the preperitoneal/transabdominal extraperitoneal arm rather than a distinct standardized named node |
| Al-Mansour 2022 | RM | **RM** | Generic retromuscular arm retained in pooled RM node |
| **Gokcal 2019** | IPOM | **rIPOM** | Standard named intraperitoneal arm |
| Gokcal 2019 | TAPP | **rTAPP** | Standard named robotic TAPP arm |
| **Kennedy 2017** | IPOM | **rIPOM** | Standard named intraperitoneal arm |
| Kennedy 2017 | TAPP | **rTAPP** | Standard named robotic TAPP arm |
| **Pacheco 2024** | TARM | **TARM** | Explicitly reported as transabdominal retromuscular approach |
| Pacheco 2024 | ETEP | **eTEP** | Explicitly reported as enhanced-view totally extraperitoneal approach |
| **Petro 2023 (REVEAL 1-year)** | ETEP | **eTEP** | Randomized named eTEP arm |
| Petro 2023 (REVEAL 1-year) | IPOM | **rIPOM** | Randomized named rIPOM arm |
| **Pini 2024** | ETEP | **eTEP** | Explicitly reported eTEP arm |
| Pini 2024 | ETAPP | **eTAPP** | Explicitly reported eTAPP arm |
| **Saleh 2025** | IPOM | **rIPOM** | Standard named intraperitoneal arm |
| Saleh 2025 | ETEP | **eTEP** | Explicitly reported eTEP arm |
| **Zaman 2023** | ETEP | **eTEP** | Explicitly reported eTEP arm |
| Zaman 2023 | TA-SM | **TA-SM** | Explicitly reported transabdominal sublay mesh arm |
| **Petro 2022 (REVEAL 30-day)** | IPOM | **rIPOM** | Randomized named rIPOM arm |
| Petro 2022 (REVEAL 30-day) | ETEP | **eTEP** | Randomized named eTEP arm |

* Denotes nodes requiring sensitivity analysis due to heterogeneity in technique definition. rIPOM = robotic intraperitoneal onlay mesh; rTAPP = robotic transabdominal preperitoneal; PP = pooled preperitoneal; RM = pooled retromuscular; eTEP = enhanced-view totally extraperitoneal; eTAPP = enhanced-view transabdominal preperitoneal.

**PRISMA 2020 Checklist**

| **Section and Topic** | **Item #** | **Checklist item** | **Location where item is reported** |
| --- | --- | --- | --- |
| **TITLE** | | | 1 |
| Title | 1 | Identify the report as a systematic review. | 1 |
| **ABSTRACT** | | | 2 |
| Abstract | 2 | See the PRISMA 2020 for Abstracts checklist. | 2 |
| **INTRODUCTION** | | | 2 |
| Rationale | 3 | Describe the rationale for the review in the context of existing knowledge. | 3 |
| Objectives | 4 | Provide an explicit statement of the objective(s) or question(s) the review addresses. | 3 |
| **METHODS** | | | 3 |
| Eligibility criteria | 5 | Specify the inclusion and exclusion criteria for the review and how studies were grouped for the syntheses. | 4 |
| Information sources | 6 | Specify all databases, registers, websites, organisations, reference lists and other sources searched or consulted to identify studies. Specify the date when each source was last searched or consulted. | 4 |
| Search strategy | 7 | Present the full search strategies for all databases, registers and websites, including any filters and limits used. | 4 |
| Selection process | 8 | Specify the methods used to decide whether a study met the inclusion criteria of the review, including how many reviewers screened each record and each report retrieved, whether they worked independently, and if applicable, details of automation tools used in the process. | 4 |
| Data collection process | 9 | Specify the methods used to collect data from reports, including how many reviewers collected data from each report, whether they worked independently, any processes for obtaining or confirming data from study investigators, and if applicable, details of automation tools used in the process. | 4 |
| Data items | 10a | List and define all outcomes for which data were sought. Specify whether all results that were compatible with each outcome domain in each study were sought (e.g. for all measures, time points, analyses), and if not, the methods used to decide which results to collect. | 4 |
|  | 10b | List and define all other variables for which data were sought (e.g. participant and intervention characteristics, funding sources). Describe any assumptions made about any missing or unclear information. | 4 |
| Study risk of bias assessment | 11 | Specify the methods used to assess risk of bias in the included studies, including details of the tool(s) used, how many reviewers assessed each study and whether they worked independently, and if applicable, details of automation tools used in the process. | 4 |
| Effect measures | 12 | Specify for each outcome the effect measure(s) (e.g. risk ratio, mean difference) used in the synthesis or presentation of results. | 5 |
| Synthesis methods | 13a | Describe the processes used to decide which studies were eligible for each synthesis (e.g. tabulating the study intervention characteristics and comparing against the planned groups for each synthesis (item #5)). | 5 |
|  | 13b | Describe any methods required to prepare the data for presentation or synthesis, such as handling of missing summary statistics, or data conversions. | 5 |
|  | 13c | Describe any methods used to tabulate or visually display results of individual studies and syntheses. | 5 |
|  | 13d | Describe any methods used to synthesize results and provide a rationale for the choice(s). If meta-analysis was performed, describe the model(s), method(s) to identify the presence and extent of statistical heterogeneity, and software package(s) used. | 5 |
|  | 13e | Describe any methods used to explore possible causes of heterogeneity among study results (e.g. subgroup analysis, meta-regression). | 5 |
|  | 13f | Describe any sensitivity analyses conducted to assess robustness of the synthesized results. | 5 |
| Reporting bias assessment | 14 | Describe any methods used to assess risk of bias due to missing results in a synthesis (arising from reporting biases). | 5 |
| Certainty assessment | 15 | Describe any methods used to assess certainty (or confidence) in the body of evidence for an outcome. | 5 |
| **RESULTS** | | | 6 |
| Study selection | 16a | Describe the results of the search and selection process, from the number of records identified in the search to the number of studies included in the review, ideally using a flow diagram. | 6 |
|  | 16b | Cite studies that might appear to meet the inclusion criteria, but which were excluded, and explain why they were excluded. | 6 |
| Study characteristics | 17 | Cite each included study and present its characteristics. | 6 |
| Risk of bias in studies | 18 | Present assessments of risk of bias for each included study. | 6 |
| Results of individual studies | 19 | For all outcomes, present, for each study: (a) summary statistics for each group (where appropriate) and (b) an effect estimate and its precision (e.g. confidence/credible interval), ideally using structured tables or plots. | 6 |
| Results of syntheses | 20a | For each synthesis, briefly summarise the characteristics and risk of bias among contributing studies. | 7 |
|  | 20b | Present results of all statistical syntheses conducted. If meta-analysis was done, present for each the summary estimate and its precision (e.g. confidence/credible interval) and measures of statistical heterogeneity. If comparing groups, describe the direction of the effect. | 7 |
|  | 20c | Present results of all investigations of possible causes of heterogeneity among study results. | 7 |
|  | 20d | Present results of all sensitivity analyses conducted to assess the robustness of the synthesized results. | 7 |
| Reporting biases | 21 | Present assessments of risk of bias due to missing results (arising from reporting biases) for each synthesis assessed. | 6 |
| Certainty of evidence | 22 | Present assessments of certainty (or confidence) in the body of evidence for each outcome assessed. | 6 |
| **DISCUSSION** | | | 8 |
| Discussion | 23a | Provide a general interpretation of the results in the context of other evidence. | 8 |
|  | 23b | Discuss any limitations of the evidence included in the review. | 9 |
|  | 23c | Discuss any limitations of the review processes used. | 9 |
|  | 23d | Discuss implications of the results for practice, policy, and future research. | 9 |
| **OTHER INFORMATION** | | | 10 |
| Registration and protocol | 24a | Provide registration information for the review, including register name and registration number, or state that the review was not registered. | 3 |
|  | 24b | Indicate where the review protocol can be accessed, or state that a protocol was not prepared. | 3 |
|  | 24c | Describe and explain any amendments to information provided at registration or in the protocol. | 3 |
| Support | 25 | Describe sources of financial or non-financial support for the review, and the role of the funders or sponsors in the review. | 12 |
| Competing interests | 26 | Declare any competing interests of review authors. | 12 |
| Availability of data, code and other materials | 27 | Report which of the following are publicly available and where they can be found: template data collection forms; data extracted from included studies; data used for all analyses; analytic code; any other materials used in the review. | 12 |

**Database Search Strategies**

| **Database** | **Search build** | **Results** |
| --- | --- | --- |
| PubMed | ( ( "Subcutaneous OnLay endoscopic Approach" OR "Subcutaneous Onlay Laparoscopic Approach" OR "Subcutaneous Onlay Laparoscopic" OR "Subcutaneous OnLay endoscopic" OR "Subcutaneous OnLay laparoscopic approach" OR "Laparoscopic Subcutaneous Onlay Mesh Repair" OR "Endoscopically Assisted Subcutaneous Onlay Mesh Repair" OR "Endoscopic Subcutaneous Onlay Repair" OR "endoscopic Subcutaneous Onlay Repair" OR "endoscopic Subcutaneous Onlay approach" OR "assisted endoscopic onlay repair" OR "Subcutaneous Onlay Mesh Repair" OR " subcutaneous video-endoscopic repair" OR "subcutaneous repair" OR "Subcutaneous mesh" OR scola OR scom ) OR ( "Laparoscopic transabdominal preperitoneal" OR "transabdominal pre-peritoneal hernia repair" OR "Transabdominal pre-peritoneal" OR "Transabdominal preperitoneal" OR "trans-abdominal pre-peritoneal" OR "Laparoscopic transperitoneal hernia repair" OR "transperitoneal repair" OR "trans-abdominal preperitoneal" OR "robotic ventral Transabdominal Pre-Peritoneal patch repair" OR "Transabdominal Preperitoneal Patch Repair" OR "transabdominal preperitoneal patch plasty" OR "Trans-Abdominal Pre-Peritoneal" OR "transabdominal preperitoneal hernioplasty" OR "Robotic TAPP" OR "laparoscopic TAPP" OR rv-tapp OR r-tapp OR tapp OR sils-tapp OR ventral-tapp ) OR ( "Laparoscopic Trans-Abdominal Retromuscular" OR "Transabdominal Retromuscular Ventral Hernia Mesh Repair" OR "Transabdominal Retromuscular" OR "Trans-abdominal Retro-muscular" OR "Trans-abdominal Retromuscular" OR " transabdominal" OR " rans-abdominal retro-muscular approach" OR "Robotic trans-abdominal retromuscular hernia repair" OR r-tarm OR tarm ) OR ( "e-TEP" OR "eTEP" OR "eRS" OR "eTEP-RS" OR etep OR "extraperitoneal" OR "endoscopic sublay repair" OR "enhanced view totally extraperitoneal" OR "totally extraperitoneal" OR "extraperitoneal repair" OR " extraperitoneal approach" OR "extraperitoneal plasty" ) OR ( "intraperitoneal onlay mesh" OR "intraperitoneal onlay mesh plus" OR ipom OR "intraperitoneal onlay" OR "intraperitoneal onlay" OR "intraperitoneal repair" OR "IPOM+" OR "IPOM plus" OR "IPOM-plus" ) ) AND ( "Herniorrhaphies" OR "Hernia Repair" OR "Hernia Repairs" OR "Repair, Hernia" OR "Repairs, Hernia" OR "Hernioplasty" OR "Hernioplasties" OR hernia ) | 4231 |
| WEB OF SCIENCE | (((((ALL=("Subcutaneous OnLay endoscopic Approach" OR "Subcutaneous Onlay Laparoscopic Approach" OR "Subcutaneous Onlay Laparoscopic" OR "Subcutaneous OnLay endoscopic" OR "Subcutaneous OnLay laparoscopic approach" OR "Laparoscopic Subcutaneous Onlay Mesh Repair" OR "Endoscopically Assisted Subcutaneous Onlay Mesh Repair" OR "Endoscopic Subcutaneous Onlay Repair" OR "endoscopic Subcutaneous Onlay Repair" OR "endoscopic Subcutaneous Onlay approach" OR "assisted endoscopic onlay repair" OR "Subcutaneous Onlay Mesh Repair" OR " subcutaneous video-endoscopic repair" OR "subcutaneous repair" OR "Subcutaneous mesh" OR SCOLA OR SCOM )) OR ALL=("Laparoscopic transabdominal preperitoneal" OR "transabdominal pre-peritoneal hernia repair" OR "Transabdominal pre-peritoneal" OR "Transabdominal preperitoneal" OR "trans-abdominal pre-peritoneal" OR "Laparoscopic transperitoneal hernia repair" OR "transperitoneal repair" OR "trans-abdominal preperitoneal" OR "robotic ventral Transabdominal Pre-Peritoneal patch repair" OR "Transabdominal Preperitoneal Patch Repair" OR "transabdominal preperitoneal patch plasty" OR "Trans-Abdominal Pre-Peritoneal" OR "transabdominal preperitoneal hernioplasty" OR "Robotic TAPP" OR "laparoscopic TAPP" OR rv-TAPP OR r-TAPP OR TAPP OR SILS-TAPP OR Ventral-TAPP )) OR ALL=("Laparoscopic Trans-Abdominal Retromuscular" OR "Transabdominal Retromuscular Ventral Hernia Mesh Repair" OR "Transabdominal Retromuscular" OR "Trans-abdominal Retro-muscular" OR "Trans-abdominal Retromuscular" OR " transabdominal" OR " rans-abdominal retro-muscular approach" OR "Robotic trans-abdominal retromuscular hernia repair" OR r-TARM OR TARM )) OR ALL=("e-TEP" OR "eTEP" OR "eRS" OR "eTEP-RS" OR etep OR "extraperitoneal" OR "endoscopic sublay repair" OR "enhanced view totally extraperitoneal" OR "totally extraperitoneal" OR "extraperitoneal repair" OR " extraperitoneal approach" OR "extraperitoneal plasty")) OR ALL=("intraperitoneal onlay mesh" OR "intraperitoneal onlay mesh plus" OR ipom OR "intraperitoneal onlay" OR "intraperitoneal onlay" OR "intraperitoneal repair" OR "IPOM+" OR "IPOM plus" OR "IPOM-plus" )) AND ALL=(“Herniorrhaphies” OR “Hernia Repair” OR “Hernia Repairs” OR “Repair, Hernia” OR “Repairs, Hernia” OR “Hernioplasty” OR “Hernioplasties” OR hernia ) | 4663 |
| Scopus | ( ( "Subcutaneous OnLay endoscopic Approach" OR "Subcutaneous Onlay Laparoscopic Approach" OR "Subcutaneous Onlay Laparoscopic" OR "Subcutaneous OnLay endoscopic" OR "Subcutaneous OnLay laparoscopic approach" OR "Laparoscopic Subcutaneous Onlay Mesh Repair" OR "Endoscopically Assisted Subcutaneous Onlay Mesh Repair" OR "Endoscopic Subcutaneous Onlay Repair" OR "endoscopic Subcutaneous Onlay Repair" OR "endoscopic Subcutaneous Onlay approach" OR "assisted endoscopic onlay repair" OR "Subcutaneous Onlay Mesh Repair" OR " subcutaneous video-endoscopic repair" OR "subcutaneous repair" OR "Subcutaneous mesh" OR scola OR scom ) OR ( "Laparoscopic transabdominal preperitoneal" OR "transabdominal pre-peritoneal hernia repair" OR "Transabdominal pre-peritoneal" OR "Transabdominal preperitoneal" OR "trans-abdominal pre-peritoneal" OR "Laparoscopic transperitoneal hernia repair" OR "transperitoneal repair" OR "trans-abdominal preperitoneal" OR "robotic ventral Transabdominal Pre-Peritoneal patch repair" OR "Transabdominal Preperitoneal Patch Repair" OR "transabdominal preperitoneal patch plasty" OR "Trans-Abdominal Pre-Peritoneal" OR "transabdominal preperitoneal hernioplasty" OR "Robotic TAPP" OR "laparoscopic TAPP" OR rv-tapp OR r-tapp OR tapp OR sils-tapp OR ventral-tapp ) OR ( "Laparoscopic Trans-Abdominal Retromuscular" OR "Transabdominal Retromuscular Ventral Hernia Mesh Repair" OR "Transabdominal Retromuscular" OR "Trans-abdominal Retro-muscular" OR "Trans-abdominal Retromuscular" OR " transabdominal" OR " rans-abdominal retro-muscular approach" OR "Robotic trans-abdominal retromuscular hernia repair" OR r-tarm OR tarm ) OR ( "e-TEP" OR "eTEP" OR "eRS" OR "eTEP-RS" OR etep OR "extraperitoneal" OR "endoscopic sublay repair" OR "enhanced view totally extraperitoneal" OR "totally extraperitoneal" OR "extraperitoneal repair" OR " extraperitoneal approach" OR "extraperitoneal plasty" ) OR ( "intraperitoneal onlay mesh" OR "intraperitoneal onlay mesh plus" OR ipom OR "intraperitoneal onlay" OR "intraperitoneal onlay" OR "intraperitoneal repair" OR "IPOM+" OR "IPOM plus" OR "IPOM-plus" ) ) AND ( "Herniorrhaphies" OR "Hernia Repair" OR "Hernia Repairs" OR "Repair, Hernia" OR "Repairs, Hernia" OR "Hernioplasty" OR "Hernioplasties" OR hernia ) | 5036 |
| embase | ( ( "Subcutaneous OnLay endoscopic Approach" OR "Subcutaneous Onlay Laparoscopic Approach" OR "Subcutaneous Onlay Laparoscopic" OR "Subcutaneous OnLay endoscopic" OR "Subcutaneous OnLay laparoscopic approach" OR "Laparoscopic Subcutaneous Onlay Mesh Repair" OR "Endoscopically Assisted Subcutaneous Onlay Mesh Repair" OR "Endoscopic Subcutaneous Onlay Repair" OR "endoscopic Subcutaneous Onlay Repair" OR "endoscopic Subcutaneous Onlay approach" OR "assisted endoscopic onlay repair" OR "Subcutaneous Onlay Mesh Repair" OR " subcutaneous video-endoscopic repair" OR "subcutaneous repair" OR "Subcutaneous mesh" OR scola OR scom ) OR ( "Laparoscopic transabdominal preperitoneal" OR "transabdominal pre-peritoneal hernia repair" OR "Transabdominal pre-peritoneal" OR "Transabdominal preperitoneal" OR "trans-abdominal pre-peritoneal" OR "Laparoscopic transperitoneal hernia repair" OR "transperitoneal repair" OR "trans-abdominal preperitoneal" OR "robotic ventral Transabdominal Pre-Peritoneal patch repair" OR "Transabdominal Preperitoneal Patch Repair" OR "transabdominal preperitoneal patch plasty" OR "Trans-Abdominal Pre-Peritoneal" OR "transabdominal preperitoneal hernioplasty" OR "Robotic TAPP" OR "laparoscopic TAPP" OR rv-tapp OR r-tapp OR tapp OR sils-tapp OR ventral-tapp ) OR ( "Laparoscopic Trans-Abdominal Retromuscular" OR "Transabdominal Retromuscular Ventral Hernia Mesh Repair" OR "Transabdominal Retromuscular" OR "Trans-abdominal Retro-muscular" OR "Trans-abdominal Retromuscular" OR " transabdominal" OR " rans-abdominal retro-muscular approach" OR "Robotic trans-abdominal retromuscular hernia repair" OR r-tarm OR tarm ) OR ( "e-TEP" OR "eTEP" OR "eRS" OR "eTEP-RS" OR etep OR "extraperitoneal" OR "endoscopic sublay repair" OR "enhanced view totally extraperitoneal" OR "totally extraperitoneal" OR "extraperitoneal repair" OR " extraperitoneal approach" OR "extraperitoneal plasty" ) OR ( "intraperitoneal onlay mesh" OR "intraperitoneal onlay mesh plus" OR ipom OR "intraperitoneal onlay" OR "intraperitoneal onlay" OR "intraperitoneal repair" OR "IPOM+" OR "IPOM plus" OR "IPOM-plus" ) ) AND ( "Herniorrhaphies" OR "Hernia Repair" OR "Hernia Repairs" OR "Repair, Hernia" OR "Repairs, Hernia" OR "Hernioplasty" OR "Hernioplasties" OR hernia ) | 6732 |
| COCHRANE | ( ( "Subcutaneous OnLay endoscopic Approach" OR "Subcutaneous Onlay Laparoscopic Approach" OR "Subcutaneous Onlay Laparoscopic" OR "Subcutaneous OnLay endoscopic" OR "Subcutaneous OnLay laparoscopic approach" OR "Laparoscopic Subcutaneous Onlay Mesh Repair" OR "Endoscopically Assisted Subcutaneous Onlay Mesh Repair" OR "Endoscopic Subcutaneous Onlay Repair" OR "endoscopic Subcutaneous Onlay Repair" OR "endoscopic Subcutaneous Onlay approach" OR "assisted endoscopic onlay repair" OR "Subcutaneous Onlay Mesh Repair" OR " subcutaneous video-endoscopic repair" OR "subcutaneous repair" OR "Subcutaneous mesh" OR scola OR scom ) OR ( "Laparoscopic transabdominal preperitoneal" OR "transabdominal pre-peritoneal hernia repair" OR "Transabdominal pre-peritoneal" OR "Transabdominal preperitoneal" OR "trans-abdominal pre-peritoneal" OR "Laparoscopic transperitoneal hernia repair" OR "transperitoneal repair" OR "trans-abdominal preperitoneal" OR "robotic ventral Transabdominal Pre-Peritoneal patch repair" OR "Transabdominal Preperitoneal Patch Repair" OR "transabdominal preperitoneal patch plasty" OR "Trans-Abdominal Pre-Peritoneal" OR "transabdominal preperitoneal hernioplasty" OR "Robotic TAPP" OR "laparoscopic TAPP" OR rv-tapp OR r-tapp OR tapp OR sils-tapp OR ventral-tapp ) OR ( "Laparoscopic Trans-Abdominal Retromuscular" OR "Transabdominal Retromuscular Ventral Hernia Mesh Repair" OR "Transabdominal Retromuscular" OR "Trans-abdominal Retro-muscular" OR "Trans-abdominal Retromuscular" OR " transabdominal" OR " rans-abdominal retro-muscular approach" OR "Robotic trans-abdominal retromuscular hernia repair" OR r-tarm OR tarm ) OR ( "e-TEP" OR "eTEP" OR "eRS" OR "eTEP-RS" OR etep OR "extraperitoneal" OR "endoscopic sublay repair" OR "enhanced view totally extraperitoneal" OR "totally extraperitoneal" OR "extraperitoneal repair" OR " extraperitoneal approach" OR "extraperitoneal plasty" ) OR ( "intraperitoneal onlay mesh" OR "intraperitoneal onlay mesh plus" OR ipom OR "intraperitoneal onlay" OR "intraperitoneal onlay" OR "intraperitoneal repair" OR "IPOM+" OR "IPOM plus" OR "IPOM-plus" ) ) AND ( "Herniorrhaphies" OR "Hernia Repair" OR "Hernia Repairs" OR "Repair, Hernia" OR "Repairs, Hernia" OR "Hernioplasty" OR "Hernioplasties" OR hernia ) | 935 |
| **Total** |  | 10621 |
| **Date** : | |  |

**3- Rankogram**

- **Leguae Tables:** The estimates from pairwise meta-analyses are situated above the diagonal line, while the estimates from the network meta-analyses are located below the diagonal line.

**1-Hernia recurrence rate**

| eTEP | . | 0.77 [0.43; 1.38] | . | . | . | . | . | . | . | 0.38 [0.09; 1.61] | 0.55 [0.28; 1.08] |
| --- | --- | --- | --- | --- | --- | --- | --- | --- | --- | --- | --- |
| 1.54 [0.84; 2.80] | PP | 0.50 [0.43; 0.58] | 0.47 [0.39; 0.56] | . | . | . | . | . | . | . | . |
| 0.77 [0.43; 1.38] | 0.50 [0.43; 0.58] | rIPOM | 0.94 [0.82; 1.07] | 0.74 [0.23; 2.43] | 2.14 [1.25; 3.64] | . | 1.81 [1.17; 2.82] | 0.40 [0.29; 0.53] | 1.25 [0.71; 2.20] | . | . |
| 0.72 [0.40; 1.31] | 0.47 [0.39; 0.56] | 0.94 [0.82; 1.07] | RM | . | . | . | . | . | . | . | . |
| 0.60 [0.20; 1.86] | 0.39 [0.15; 1.04] | 0.79 [0.30; 2.06] | 0.84 [0.32; 2.22] | rRM | . | . | 2.15 [0.59; 7.91] | . | . | . | . |
| 1.54 [0.70; 3.36] | 1.00 [0.58; 1.73] | 2.00 [1.18; 3.38] | 2.13 [1.24; 3.66] | 2.54 [0.86; 7.51] | rRS | . | 1.17 [0.59; 2.30] | 0.19 [0.11; 0.30] | . | . | . |
| 0.91 [0.37; 2.21] | 0.59 [0.30; 1.17] | 1.18 [0.60; 2.31] | 1.26 [0.63; 2.49] | 1.50 [0.46; 4.87] | 0.59 [0.25; 1.39] | rTA-RM | . | . | 1.06 [0.74; 1.53] | . | . |
| 1.39 [0.67; 2.89] | 0.91 [0.57; 1.44] | 1.81 [1.17; 2.82] | 1.93 [1.22; 3.06] | 2.31 [0.87; 6.13] | 0.91 [0.49; 1.69] | 1.54 [0.69; 3.44] | rTAPP | 0.16 [0.09; 0.27] | . | . | . |
| 0.28 [0.15; 0.54] | 0.18 [0.13; 0.26] | 0.37 [0.28; 0.49] | 0.39 [0.29; 0.54] | 0.47 [0.17; 1.26] | 0.19 [0.11; 0.30] | 0.31 [0.15; 0.65] | 0.20 [0.13; 0.32] | rTAR | . | . | . |
| 0.96 [0.43; 2.16] | 0.63 [0.35; 1.12] | 1.25 [0.71; 2.20] | 1.33 [0.75; 2.38] | 1.59 [0.52; 4.87] | 0.63 [0.29; 1.36] | 1.06 [0.74; 1.53] | 0.69 [0.34; 1.41] | 3.39 [1.79; 6.39] | rTEP-RM | . | . |
| 0.38 [0.09; 1.61] | 0.25 [0.05; 1.18] | 0.49 [0.10; 2.34] | 0.53 [0.11; 2.51] | 0.63 [0.10; 3.93] | 0.25 [0.05; 1.28] | 0.42 [0.08; 2.28] | 0.27 [0.05; 1.37] | 1.33 [0.27; 6.51] | 0.39 [0.08; 2.07] | TA-SM | . |
| 0.55 [0.28; 1.08] | 0.36 [0.15; 0.88] | 0.72 [0.30; 1.74] | 0.77 [0.31; 1.88] | 0.92 [0.25; 3.40] | 0.36 [0.13; 1.01] | 0.61 [0.20; 1.86] | 0.40 [0.15; 1.07] | 1.95 [0.77; 4.95] | 0.58 [0.20; 1.65] | 1.46 [0.30; 7.18] | TARM |

| eTAPP | 0.30 [0.02; 5.99] | . | . | . | . | . | . | . | . | . | . |
| --- | --- | --- | --- | --- | --- | --- | --- | --- | --- | --- | --- |
| 0.30 [0.02; 5.99] | eTEP | . | 1.14 [0.63; 2.07] | . | . | . | . | 0.60 [0.04; 9.30] | . | . | 1.33 [0.59; 3.00] |
| 0.49 [0.02; 10.30] | 1.62 [0.88; 2.97] | PP | 0.67 [0.55; 0.81] | 0.44 [0.35; 0.55] | . | . | . | . | . | . | . |
| 0.33 [0.02; 6.86] | 1.08 [0.61; 1.93] | 0.67 [0.55; 0.81] | rIPOM | 0.65 [0.55; 0.78] | 1.35 [0.61; 2.99] | 1.55 [0.90; 2.67] | . | 1.82 [1.30; 2.56] | 0.60 [0.40; 0.89] | 4.43 [2.10; 9.36] | . |
| 0.21 [0.01; 4.51] | 0.71 [0.39; 1.30] | 0.44 [0.35; 0.55] | 0.65 [0.55; 0.78] | RM | . | . | . | . | . | . | . |
| 0.43 [0.02; 9.71] | 1.41 [0.56; 3.55] | 0.87 [0.41; 1.85] | 1.30 [0.63; 2.69] | 1.99 [0.94; 4.19] | rRM | . | . | 1.80 [0.70; 4.64] | . | . | . |
| 0.52 [0.02; 11.34] | 1.71 [0.78; 3.73] | 1.06 [0.60; 1.85] | 1.58 [0.93; 2.67] | 2.41 [1.39; 4.20] | 1.22 [0.50; 2.94] | rRS | . | 1.08 [0.57; 2.07] | 0.38 [0.23; 0.64] | . | . |
| 0.51 [0.02; 12.53] | 1.70 [0.55; 5.26] | 1.05 [0.39; 2.83] | 1.57 [0.60; 4.14] | 2.40 [0.90; 6.43] | 1.21 [0.36; 4.05] | 0.99 [0.33; 2.99] | rTA-RM | . | . | 2.82 [1.52; 5.22] | . |
| 0.58 [0.03; 12.44] | 1.93 [1.00; 3.74] | 1.19 [0.81; 1.76] | 1.78 [1.27; 2.50] | 2.72 [1.86; 3.98] | 1.37 [0.65; 2.90] | 1.13 [0.64; 1.99] | 1.13 [0.41; 3.17] | rTAPP | 0.35 [0.21; 0.60] | . | . |
| 0.20 [0.01; 4.25] | 0.66 [0.33; 1.31] | 0.41 [0.27; 0.62] | 0.61 [0.41; 0.89] | 0.93 [0.61; 1.41] | 0.47 [0.21; 1.04] | 0.38 [0.23; 0.64] | 0.39 [0.14; 1.09] | 0.34 [0.22; 0.52] | rTAR | . | . |
| 1.45 [0.06; 33.25] | 4.79 [1.86; 12.34] | 2.97 [1.37; 6.43] | 4.43 [2.10; 9.36] | 6.76 [3.14; 14.59] | 3.40 [1.20; 9.65] | 2.80 [1.12; 6.99] | 2.82 [1.52; 5.22] | 2.48 [1.09; 5.64] | 7.31 [3.16; 16.91] | rTEP-RM | . |
| 0.40 [0.02; 8.89] | 1.33 [0.59; 3.00] | 0.82 [0.30; 2.28] | 1.23 [0.45; 3.34] | 1.88 [0.68; 5.17] | 0.94 [0.28; 3.24] | 0.78 [0.25; 2.40] | 0.78 [0.19; 3.14] | 0.69 [0.24; 1.97] | 2.03 [0.70; 5.89] | 0.28 [0.08; 0.97] | TA-SM |

**2-Overall complication rate**

**3- Overall SSI 1**

| PP | 0.85 [0.40; 1.81] | 0.67 [0.25; 1.78] | . | . | . | . | . | . |
| --- | --- | --- | --- | --- | --- | --- | --- | --- |
| 0.85 [0.40; 1.81] | rIPOM | 0.78 [0.34; 1.80] | 3.19 [0.60; 17.02] | 8.54 [1.12; 65.45] | . | 3.18 [1.29; 7.84] | 1.10 [0.44; 2.77] | 6.00 [0.74; 48.52] |
| 0.67 [0.25; 1.78] | 0.78 [0.34; 1.80] | RM | . | . | . | . | . | . |
| 2.57 [0.43; 15.40] | 3.03 [0.60; 15.35] | 3.85 [0.62; 23.90] | rRM | . | . | 1.00 [0.11; 9.16] | . | . |
| 7.38 [0.85; 64.31] | 8.70 [1.14; 66.09] | 11.08 [1.24; 99.20] | 2.88 [0.22; 38.06] | rRS | . | 0.34 [0.04; 3.23] | 0.13 [0.02; 1.03] | . |
| 1.82 [0.16; 20.60] | 2.14 [0.21; 21.50] | 2.73 [0.24; 31.68] | 0.71 [0.04; 11.89] | 0.25 [0.01; 5.31] | rTA-RM | . | . | 2.80 [1.06; 7.42] |
| 2.69 [0.83; 8.71] | 3.17 [1.29; 7.79] | 4.03 [1.18; 13.74] | 1.05 [0.18; 6.04] | 0.36 [0.04; 3.10] | 1.48 [0.12; 17.57] | rTAPP | 0.38 [0.10; 1.44] | . |
| 0.95 [0.29; 3.09] | 1.12 [0.45; 2.77] | 1.43 [0.42; 4.88] | 0.37 [0.06; 2.33] | 0.13 [0.02; 1.03] | 0.52 [0.04; 6.23] | 0.35 [0.11; 1.10] | rTAR | . |
| 5.09 [0.55; 47.03] | 6.00 [0.74; 48.52] | 7.64 [0.81; 72.49] | 1.98 [0.14; 27.99] | 0.69 [0.04; 12.69] | 2.80 [1.06; 7.42] | 1.90 [0.19; 18.45] | 5.35 [0.55; 52.20] | rTEP-RM |

**4- Overall SSI 2**

| eTEP | 0.72 [0.08; 6.75] | 0.20 [0.01; 4.81] |
| --- | --- | --- |
| 0.72 [0.08; 6.75] | TA-SM | . |
| 0.20 [0.01; 4.81] | 0.28 [0.01; 13.58] | TARM |

**5- Postoperative Pain Score**

| eTEP | . | -0.12 (-0.61; 0.38) | . | . | . | . | -0.48 (-1.34; 0.39) |
| --- | --- | --- | --- | --- | --- | --- | --- |
| 0.41 (-0.46; 1.28) | PP | -0.35 (-1.11; 0.42) | -0.24 (-1.01; 0.52) | . | . | . | . |
| -0.12 (-0.61; 0.38) | -0.53 (-1.24; 0.19) | rIPOM | 0.39 (-0.17; 0.95) | . | 0.33 (-0.24; 0.90) | -0.35 (-1.18; 0.48) | . |
| 0.35 (-0.39; 1.08) | -0.06 (-0.77; 0.65) | 0.47 (-0.07; 1.01) | rRM | . | -0.63 (-1.45; 0.19) | . | . |
| -0.47 (-1.73; 0.80) | -0.87 (-2.24; 0.49) | -0.35 (-1.51; 0.82) | -0.81 (-2.10; 0.47) | rTA-RM | . | 0.00 (-0.82; 0.82) | . |
| 0.11 (-0.64; 0.85) | -0.30 (-1.16; 0.55) | 0.22 (-0.33; 0.78) | -0.24 (-0.90; 0.42) | 0.57 (-0.72; 1.86) | rTAPP | . | . |
| -0.47 (-1.43; 0.50) | -0.87 (-1.97; 0.22) | -0.35 (-1.18; 0.48) | -0.81 (-1.81; 0.18) | -0.00 (-0.82; 0.82) | -0.57 (-1.57; 0.42) | rTEP-RM | . |
| -0.48 (-1.34; 0.39) | -0.88 (-2.11; 0.34) | -0.36 (-1.36; 0.64) | -0.82 (-1.96; 0.31) | -0.01 (-1.55; 1.52) | -0.58 (-1.72; 0.56) | -0.01 (-1.31; 1.29) | TARM |

**6- Length of hospital stay (LOS)**

| eTAPP | -0.30 (-1.45; 0.85) | . | . | . | . | . | . |
| --- | --- | --- | --- | --- | --- | --- | --- |
| -0.30 (-1.45; 0.85) | eTEP | -0.49 (-1.01; 0.03) | . | . | . | . | -0.90 (-1.56;-0.24) |
| -0.79 (-2.05; 0.47) | -0.49 (-1.01; 0.03) | rIPOM | 0.00 (-0.05; 0.05) | 0.33 (-1.33; 1.99) | 1.00 (-0.16; 2.16) | . | . |
| -0.79 (-2.05; 0.47) | -0.49 (-1.01; 0.03) | 0.00 (-0.05; 0.05) | RM | . | . | . | . |
| -0.46 (-2.54; 1.62) | -0.16 (-1.90; 1.58) | 0.33 (-1.33; 1.99) | 0.33 (-1.33; 1.99) | rTA-RM | . | 5.00 ( 1.63; 8.37) | . |
| 0.21 (-1.51; 1.93) | 0.51 (-0.76; 1.79) | 1.00 (-0.16; 2.16) | 1.00 (-0.16; 2.16) | 0.67 (-1.36; 2.70) | rTAPP | . | . |
| 4.54 ( 0.58; 8.50) | 4.84 ( 1.05; 8.63) | 5.33 ( 1.58; 9.08) | 5.33 ( 1.58; 9.08) | 5.00 ( 1.63; 8.37) | 4.33 ( 0.40; 8.26) | rTEP-RM | . |
| -1.20 (-2.52; 0.12) | -0.90 (-1.56;-0.24) | -0.41 (-1.25; 0.43) | -0.41 (-1.25; 0.43) | -0.74 (-2.60; 1.12) | -1.41 (-2.84; 0.02) | -5.74 (-9.59;-1.89) | TA-SM |

**7- Seroma formation rate**

| eTAPP | 0.50 [0.02; 11.80] | . | . | . | . | . | . | . | . |
| --- | --- | --- | --- | --- | --- | --- | --- | --- | --- |
| 0.50 [0.02; 11.80] | eTEP | 14.42 [0.85; 245.78] | . | . | . | . | . | 6.48 [1.36; 30.99] | 0.75 [0.22; 2.61] |
| 7.26 [0.10; 504.96] | 14.42 [0.85; 245.78] | rIPOM | 2.36 [0.41; 13.45] | 1.86 [0.67; 5.18] | 1.27 [0.68; 2.35] | 0.65 [0.30; 1.37] | 2.50 [0.50; 12.44] | . | . |
| 20.48 [0.24; 1784.01] | 40.64 [1.72; 961.58] | 2.82 [0.69; 11.46] | rRM | . | 0.58 [0.13; 2.67] | . | . | . | . |
| 14.34 [0.18; 1116.62] | 28.45 [1.41; 573.49] | 1.97 [0.73; 5.30] | 0.70 [0.13; 3.71] | rRS | 0.57 [0.19; 1.65] | 0.35 [0.13; 0.95] | . | . | . |
| 8.77 [0.12; 634.28] | 17.40 [0.96; 314.81] | 1.21 [0.67; 2.16] | 0.43 [0.11; 1.71] | 0.61 [0.22; 1.68] | rTAPP | 0.61 [0.27; 1.39] | 3.67 [1.06; 12.66] | . | . |
| 4.97 [0.07; 366.66] | 9.87 [0.53; 183.61] | 0.68 [0.34; 1.39] | 0.24 [0.05; 1.11] | 0.35 [0.13; 0.95] | 0.57 [0.27; 1.19] | rTAR | . | . | . |
| 25.97 [0.33; 2050.34] | 51.54 [2.51; 1059.46] | 3.58 [1.25; 10.19] | 1.27 [0.24; 6.80] | 1.81 [0.46; 7.17] | 2.96 [1.08; 8.09] | 5.22 [1.59; 17.16] | rTEP-RM | . | . |
| 3.27 [0.10; 110.39] | 6.48 [1.36; 30.99] | 0.45 [0.02; 11.47] | 0.16 [0.00; 5.44] | 0.23 [0.01; 6.73] | 0.37 [0.01; 10.01] | 0.66 [0.02; 18.08] | 0.13 [0.00; 3.78] | TA-SM | . |
| 0.38 [0.01; 11.23] | 0.75 [0.22; 2.61] | 0.05 [0.00; 1.15] | 0.02 [0.00; 0.55] | 0.03 [0.00; 0.68] | 0.04 [0.00; 1.01] | 0.08 [0.00; 1.82] | 0.01 [0.00; 0.38] | 0.12 [0.02; 0.86] | TARM |

**8- 30-day hospital readmission rate**

| PP | 0.58 [0.38; 0.87] | 0.39 [0.24; 0.64] | . | . | . | . |
| --- | --- | --- | --- | --- | --- | --- |
| 0.58 [0.38; 0.87] | rIPOM | 0.68 [0.47; 0.98] | 0.82 [0.04; 17.16] | . | 0.87 [0.12; 6.57] | 2.00 [0.52; 7.67] |
| 0.39 [0.24; 0.64] | 0.68 [0.47; 0.98] | RM | . | . | . | . |
| 0.70 [0.06; 8.98] | 1.22 [0.10; 15.07] | 1.81 [0.14; 22.94] | rRM | . | 0.63 [0.08; 5.11] | . |
| 1.15 [0.16; 8.11] | 2.00 [0.30; 13.46] | 2.96 [0.43; 20.65] | 1.64 [0.07; 38.30] | rTA-RM | . | 1.00 [0.26; 3.86] |
| 0.50 [0.06; 3.95] | 0.87 [0.12; 6.57] | 1.29 [0.16; 10.06] | 0.71 [0.09; 5.41] | 0.43 [0.03; 7.00] | rTAPP | . |
| 1.15 [0.28; 4.70] | 2.00 [0.52; 7.67] | 2.96 [0.74; 11.94] | 1.64 [0.09; 28.25] | 1.00 [0.26; 3.86] | 2.30 [0.20; 26.10] | rTEP-RM |

**9- Reoperation rate**

| eTEP | . | 1.73 [0.24; 12.64] | . | . | . | . | . | 0.20 [0.01; 4.81] |
| --- | --- | --- | --- | --- | --- | --- | --- | --- |
| 3.86 [0.45; 33.30] | PP | 0.45 [0.19; 1.03] | 0.17 [0.07; 0.42] | . | . | . | . | . |
| 1.73 [0.24; 12.64] | 0.45 [0.19; 1.03] | rIPOM | 0.38 [0.22; 0.67] | 0.82 [0.04; 17.16] | . | 1.55 [0.04; 67.06] | 2.33 [0.35; 15.34] | . |
| 0.67 [0.09; 5.24] | 0.17 [0.07; 0.42] | 0.38 [0.22; 0.67] | RM | . | . | . | . | . |
| 1.42 [0.04; 53.72] | 0.37 [0.02; 8.62] | 0.82 [0.04; 17.16] | 2.13 [0.10; 46.82] | rRM | . | 1.89 [0.09; 41.24] | . | . |
| 0.81 [0.01; 47.69] | 0.21 [0.01; 8.11] | 0.47 [0.01; 16.41] | 1.21 [0.03; 44.46] | 0.57 [0.01; 61.67] | rTA-RM | . | 5.00 [0.24; 102.56] | . |
| 2.68 [0.04; 190.05] | 0.69 [0.01; 32.99] | 1.55 [0.04; 67.06] | 4.02 [0.09; 181.29] | 1.89 [0.09; 41.24] | 3.31 [0.02; 591.61] | rTAPP | . | . |
| 4.05 [0.26; 62.48] | 1.05 [0.13; 8.22] | 2.33 [0.35; 15.34] | 6.06 [0.85; 43.10] | 2.85 [0.08; 102.18] | 5.00 [0.24; 102.56] | 1.51 [0.02; 102.15] | rTEP-RM | . |
| 0.20 [0.01; 4.81] | 0.05 [0.00; 2.41] | 0.12 [0.00; 4.90] | 0.30 [0.01; 13.26] | 0.14 [0.00; 17.66] | 0.25 [0.00; 43.57] | 0.08 [0.00; 15.24] | 0.05 [0.00; 3.29] | TARM |

| eTAPP | 20.00 ( -16.99; 56.99) | . | . | . | . | . | . | . | . | . |
| --- | --- | --- | --- | --- | --- | --- | --- | --- | --- | --- |
| 20.00 ( -16.99; 56.99) | eTEP | 52.96 ( 38.35; 67.56) | . | . | . | . | . | . | 2.50 ( -27.94; 32.94) | 0.17 ( -20.31; 20.65) |
| 72.96 ( 33.19; 112.72) | 52.96 ( 38.35; 67.56) | rIPOM | -38.13 ( -53.92; -22.35) | -21.67 ( -36.69; -6.65) | . | 1.15 ( -6.39; 8.69) | -141.50 (-163.94;-119.06) | -16.00 ( -35.69; 3.69) | . | . |
| 37.33 ( -5.11; 79.78) | 17.33 ( -3.49; 38.16) | -35.62 ( -50.46; -20.78) | rRM | . | . | 33.82 ( 17.83; 49.80) | . | . | . | . |
| 46.63 ( 4.59; 88.67) | 26.63 ( 6.64; 46.62) | -26.32 ( -39.97; -12.68) | 9.30 ( -10.09; 28.68) | rRS | . | 31.17 ( 16.85; 45.49) | -119.83 (-142.71; -96.95) | . | . | . |
| 22.46 ( -31.49; 76.40) | 2.46 ( -36.81; 41.72) | -50.50 ( -86.95; -14.05) | -14.88 ( -54.23; 24.48) | -24.18 ( -63.09; 14.74) | rTA-RM | . | . | 34.50 ( 3.82; 65.18) | . | . |
| 74.08 ( 33.60; 114.55) | 54.08 ( 37.63; 70.52) | 1.12 ( -6.42; 8.66) | 36.74 ( 21.84; 51.65) | 27.44 ( 14.04; 40.85) | 51.62 ( 14.40; 88.84) | rTAPP | -151.00 (-172.98;-129.02) | . | . | . |
| -73.20 (-118.43; -27.97) | -93.20 (-119.22; -67.17) | -146.15 (-167.69;-124.61) | -110.53 (-136.10; -84.96) | -119.83 (-142.71; -96.95) | -95.65 (-137.99; -53.32) | -147.27 (-168.67;-125.88) | rTAR | . | . | . |
| 56.96 ( 12.58; 101.33) | 36.96 ( 12.44; 61.47) | -16.00 ( -35.69; 3.69) | 19.62 ( -5.03; 44.28) | 10.32 ( -13.63; 34.27) | 34.50 ( 3.82; 65.18) | -17.12 ( -38.20; 3.96) | 130.15 ( 100.97; 159.33) | rTEP-RM | . | . |
| 22.50 ( -25.40; 70.40) | 2.50 ( -27.94; 32.94) | -50.46 ( -84.22; -16.70) | -14.83 ( -51.71; 22.04) | -24.13 ( -60.54; 12.28) | 0.04 ( -49.64; 49.73) | -51.58 ( -86.17; -16.98) | 95.70 ( 55.65; 135.74) | -34.46 ( -73.54; 4.62) | TA-SM | . |
| 20.17 ( -22.11; 62.45) | 0.17 ( -20.31; 20.65) | -52.79 ( -77.94; -27.63) | -17.16 ( -46.37; 12.04) | -26.46 ( -55.08; 2.15) | -2.29 ( -46.57; 42.00) | -53.91 ( -80.17; -27.65) | 93.37 ( 60.25; 126.48) | -36.79 ( -68.73; -4.84) | -2.33 ( -39.01; 34.35) | TARM |

**10- Total Operative Time**

**11- Console Time**

| rIPOM | -62.00 (-116.65; -7.35) | -20.67 ( -38.64; -2.70) | . | -2.13 ( -12.54; 8.27) | -135.84 (-159.41;-112.27) | -17.33 ( -41.81; 7.15) |
| --- | --- | --- | --- | --- | --- | --- |
| -63.23 (-113.40; -13.06) | rRM | . | . | 63.00 ( 2.47; 123.53) | . | . |
| -25.94 ( -42.43; -9.45) | 37.29 ( -15.09; 89.67) | rRS | . | 28.50 ( 11.00; 46.00) | -115.17 (-139.07; -91.27) | . |
| -53.17 ( -93.44; -12.90) | 10.06 ( -54.27; 74.39) | -27.23 ( -70.75; 16.28) | rTA-RM | . | . | 35.84 ( 3.86; 67.82) |
| -2.13 ( -12.54; 8.27) | 61.10 ( 10.70; 111.50) | 23.81 ( 7.51; 40.10) | 51.04 ( 9.44; 92.63) | rTAPP | -143.67 (-166.88;-120.46) | . |
| -141.11 (-163.57;-118.65) | -77.88 (-132.44; -23.32) | -115.17 (-139.07; -91.27) | -87.94 (-134.05; -41.83) | -138.98 (-161.30;-116.65) | rTAR | . |
| -17.33 ( -41.81; 7.15) | 45.90 ( -9.92; 101.72) | 8.61 ( -20.90; 38.12) | 35.84 ( 3.86; 67.82) | -15.20 ( -41.79; 11.40) | 123.78 ( 90.56; 157.00) | rTEP-RM |

**12- Hematoma rate1**

| rIPOM | 1.47 [0.30; 7.32] | 2.33 [0.57; 9.61] | . | 2.43 [0.93; 6.34] | 1.50 [0.43; 5.25] | 3.00 [0.49; 18.47] |
| --- | --- | --- | --- | --- | --- | --- |
| 1.35 [0.30; 6.01] | rRM | . | . | 2.89 [0.32; 26.50] | . | . |
| 2.35 [0.58; 9.47] | 1.74 [0.23; 12.99] | rRS | . | 1.02 [0.18; 5.80] | 0.64 [0.13; 3.21] | . |
| 9.00 [0.23; 352.51] | 6.65 [0.13; 348.69] | 3.83 [0.08; 193.40] | rTA-RM | . | . | 0.33 [0.01; 8.06] |
| 2.47 [0.95; 6.40] | 1.82 [0.36; 9.32] | 1.05 [0.22; 4.94] | 0.27 [0.01; 12.13] | rTAPP | 0.63 [0.13; 3.15] | . |
| 1.52 [0.45; 5.15] | 1.12 [0.17; 7.48] | 0.64 [0.13; 3.21] | 0.17 [0.00; 8.05] | 0.61 [0.15; 2.49] | rTAR | . |
| 3.00 [0.49; 18.47] | 2.22 [0.21; 23.26] | 1.28 [0.13; 12.58] | 0.33 [0.01; 8.06] | 1.22 [0.16; 9.47] | 1.98 [0.22; 17.69] | rTEP-RM |

**13- Hematoma rate 2**

| eTEP | 0.54 [0.06; 4.71] | 0.75 [0.22; 2.61] |
| --- | --- | --- |
| 0.54 [0.06; 4.71] | TA-SM | . |
| 0.75 [0.22; 2.61] | 1.39 [0.11; 16.91] | TARM |

**14- Intraoperative complication rate**

| PP | 0.68 [0.31; 1.48] | 0.69 [0.24; 1.99] | . | . | . | . | . | . |
| --- | --- | --- | --- | --- | --- | --- | --- | --- |
| 0.68 [0.31; 1.48] | rIPOM | 1.02 [0.43; 2.45] | 2.45 [0.32; 18.77] | 3.11 [0.35; 27.51] | . | 3.92 [0.85; 18.04] | 0.70 [0.18; 2.75] | 7.00 [0.37; 132.97] |
| 0.69 [0.24; 1.99] | 1.02 [0.43; 2.45] | RM | . | . | . | . | . | . |
| 1.60 [0.19; 13.28] | 2.37 [0.33; 16.90] | 2.32 [0.27; 19.94] | rRM | . | . | 1.89 [0.09; 41.24] | . | . |
| 2.19 [0.22; 21.78] | 3.24 [0.37; 28.08] | 3.17 [0.31; 32.62] | 1.37 [0.08; 24.49] | rRS | . | 1.02 [0.06; 16.16] | 0.23 [0.03; 1.99] | . |
| 0.53 [0.01; 35.44] | 0.78 [0.01; 48.68] | 0.76 [0.01; 52.24] | 0.33 [0.00; 31.94] | 0.24 [0.00; 25.51] | rTA-RM | . | . | 9.00 [0.49; 164.51] |
| 2.65 [0.48; 14.73] | 3.92 [0.85; 18.04] | 3.84 [0.66; 22.31] | 1.65 [0.17; 16.28] | 1.21 [0.10; 14.22] | 5.04 [0.06; 414.52] | rTAPP | 0.22 [0.03; 1.96] | . |
| 0.49 [0.11; 2.32] | 0.73 [0.19; 2.77] | 0.72 [0.15; 3.53] | 0.31 [0.03; 3.18] | 0.23 [0.03; 1.99] | 0.94 [0.01; 72.52] | 0.19 [0.03; 1.11] | rTAR | . |
| 4.74 [0.23; 99.61] | 7.00 [0.37; 132.97] | 6.85 [0.32; 147.90] | 2.95 [0.09; 101.60] | 2.16 [0.06; 83.20] | 9.00 [0.49; 164.51] | 1.78 [0.06; 49.17] | 9.58 [0.38; 242.68] | rTEP-RM |

**supplemantary table 6. Defect Closure Status**

| **Study** | **N** | **Study Arms** | **Arm 1** | **Arm 2** | **Arm 3** | **Arm 4** |
| --- | --- | --- | --- | --- | --- | --- |
|  |  | **(Network node)** | **Defect Closure n (%)** | **Defect Closure n (%)** | **Defect Closure n (%)** | **Defect Closure n (%)** |
| **Al-Mansour 2022** | ~7,257 | *rIPOM \| PP \| RM* | 79.0%  (3,540/4,482) | 92.4%  (1,685/1,826) | 93.8%  (889/949) | — |
| **Gokcal 2019** | 208 | *rIPOM \| rTAPP* | 87.5%  (91/104) | 94.2%  (98/104) | — | — |
| **Kennedy 2017** | 63 | *rIPOM \| rTAPP* | Not tabulated  (optional per technique) | **100%**  **(technique description)** | — | — |
| **Pacheco 2024** | 96 | *TARM \| eTEP* | **100%**  **(standard technique)** | **100%**  **(standard technique)** | — | — |
| **Petro 2022 (REVEAL RCT)** | 100 | *rIPOM \| eTEP* | **100%**  **(protocol-mandated)** | **100%**  **(protocol-mandated)** | — | — |
| **Petro 2023 (REVEAL 1-yr)** | 100 | *rIPOM \| eTEP* | **100%**  **(protocol-mandated)** | **100%**  **(protocol-mandated)** | — | — |
| **Pini 2024** | 53 | *eTEP \| eTAPP* | **100%**  **(32/32)** | **100%**  **(21/21)** | — | — |
| **Saleh 2025** | 88 | *rIPOM \| eTEP* | **100%**  **(described as standard)** | **100%**  **(described as standard)** | — | — |
| **Zaman 2023** | 158 | *TA-SM \| eTEP* | **100%**  **(technique description)** | **100%**  **(technique description)** | — | — |
| **Kudsi 2020a (IPOM vs TEP-RM)** | 136 | *rIPOM \| rTEP-RM* | 79.4%  (54/68) | **100%**  **(68/68)** | — | — |
| **Kudsi 2020b (TA vs TEP retromuscular)** | 164 | *rTA-RM \| rTEP-RM* | **96.3%**  **(79/82)** | **100%**  **(82/82)** | — | — |
| **Kudsi 2021** | 269 | *rIPOM \| rTAPP \| rRM* | 87.8%  (79/90) | **96.3%**  **(104/108)** | **100%**  **(71/71)** | — |
| **Kudsi 2022 (Ann Surg)** | 644 | *rIPOM \| rTAPP \| rRS \| rTAR* | 83.8%  (165/197) | 92.3%  (144/156) | **98.7%**  **(151/153)** | **97.1%**  **(134/138)** |
| **Kudsi 2020 (lateral incisional)** | 26 | *rIPOM \| rTAPP \| rRM* | 60.0%  (3/5) | **100%**  **(8/8)** | 92.3%  (12/13) | — |

*† Three studies (Petro 2022, Petro 2023, Saleh 2025) mandated 100% closure by protocol for both arms; these are not representative of real-world rIPOM practice where closure rates in observational cohorts ranged from 60–88%. Excluding protocol-mandated studies, the weighted rIPOM closure rate across observational series is approximately 80–84%, compared with 92–100% for all preperitoneal and retromuscular approaches.*
